# Suppression of epithelial folding at actomyosin-enriched compartment boundaries downstream of Wingless signalling in *Drosophila*

**DOI:** 10.1242/dev.155325

**Published:** 2018-04-24

**Authors:** Jose M. Urbano, Huw W. Naylor, Elena Scarpa, Leila Muresan, Bénédicte Sanson

**Affiliations:** 1 Department of Physiology, Development and Neuroscience, University of Cambridge, Anatomy Building, Downing Street, Cambridge, CB2 3DY, UK; 2 Cambridge Advanced Imaging Centre, University of Cambridge, Anatomy Building, Downing Street, Cambridge, CB2 3DY, UK

**Keywords:** Apico-basal polarity, Contractility, Embryo, Epithelium, Morphogenesis, Planar polarity

## Abstract

Epithelial folding shapes embryos and tissues during development. Here, we investigate the coupling between epithelial folding and actomyosin-enriched compartmental boundaries. The mechanistic relationship between the two is unclear, because actomyosin-enriched boundaries are not necessarily associated with folds. Also, some cases of epithelial folding occur independently of actomyosin contractility. We investigated the shallow folds called parasegment grooves that form at boundaries between anterior and posterior compartments in the early *Drosophila* embryo. We demonstrate that formation of these folds requires the presence of an actomyosin enrichment along the boundary cell-cell contacts. These enrichments, which require Wingless signalling, increase interfacial tension not only at the level of the adherens junctions but also along the lateral surfaces. We find that epithelial folding is normally under inhibitory control because different genetic manipulations, including depletion of the Myosin II phosphatase Flapwing, increase the depth of folds at boundaries. Fold depth correlates with the levels of Bazooka (Baz), the Par-3 homologue, along the boundary cell-cell contacts. Moreover, Wingless and Hedgehog signalling have opposite effects on fold depth at the boundary that correlate with changes in Baz planar polarity.

## Abstract (precis)

**Highlighted Article:** In the *Drosophila* embryo, Wingless signalling is required to maintain robust actomyosin enrichment at parasegmental boundaries, and suppresses epithelial folding along these boundaries, where Hedgehog signalling has opposing effects.

## INTRODUCTION

Epithelial sheet bending is essential to elaborate the anatomy of animal bodies. It is ubiquitous throughout development, from gastrulation to organogenesis (Bazin-Lopez et al., 2015; Keller and Shook, 2011). The mechanisms identified so far that promote epithelial sheet bending are diverse (Pearl et al., 2017). One of the best-studied mechanisms is apical constriction mediated by actomyosin activation at the apical end of epithelial cells (Martin and Goldstein, 2014). However, not all mechanisms uncovered for epithelial folding require actomyosin activity; a basal shift of the adherens junctions (AJs) is required instead for dorsal fold formation in *Drosophila* gastrulae (Wang et al., 2012).

Here, we investigate the relationship between epithelial folding and planar actomyosin cables that are often found in developing epithelia (Fagotto, 2015; Monier et al., 2011; Röper, 2013). For example, actomyosin enrichments are found at the level of AJs at compartmental boundaries in *Drosophila* epithelial tissues, forming supracellular contractile cables that are required for lineage restriction (Landsberg et al., 2009; Monier et al., 2010). In some instances, such as in segments, actomyosin-rich cables are associated with folds (Calzolari et al., 2014; Mulinari et al., 2008). In other cases, such as for the anteroposterior (AP) compartmental boundary in *Drosophila* wing discs, actomyosin-rich boundaries are anatomically ‘silent’, with no folding observed (Landsberg et al., 2009). Intriguingly, however, some mutant backgrounds can generate a fold along the AP boundary in wing discs, suggesting that fold formation is normally suppressed at this compartmental boundary (Liu et al., 2016; Shen et al., 2008).

In *Drosophila* embryos, the AP compartmental boundaries (called parasegmental boundaries, PSBs) first enrich actomyosin at gastrulation, during germ-band extension (Tetley et al., 2016). Once the germ-band is extended, Wingless signalling on one side of the compartmental boundary maintains these enrichments, which act as mechanical barriers to keep dividing cells in their compartment of origin (Monier et al., 2010). No epithelial folding is associated with actomyosin-enriched PSBs during gastrulation, and it is only 2 h later, during germ-band extended stages, that shallow indentations form, the parasegmental folds or grooves (Larsen et al., 2008; Martinez-Arias, 1993; Martinez-Arias and Lawrence, 1985). Here, we first establish that actomyosin contractility is required for parasegment fold formation. Next, we provide evidence that fold formation is normally suppressed at PSBs, under the control of Wingless signalling. This moderation of fold formation at PSBs requires Myosin II phosphatase activity and also correlates with the depletion of Bazooka, the homologue of vertebrate Par-3. This shows that specific cellular mechanisms suppress fold formation at actomyosin-rich boundaries.

## RESULTS

### Wingless signalling maintains earlier planar polarities specifically at the PSBs at germ-band extended stages

**Fig. 1.**
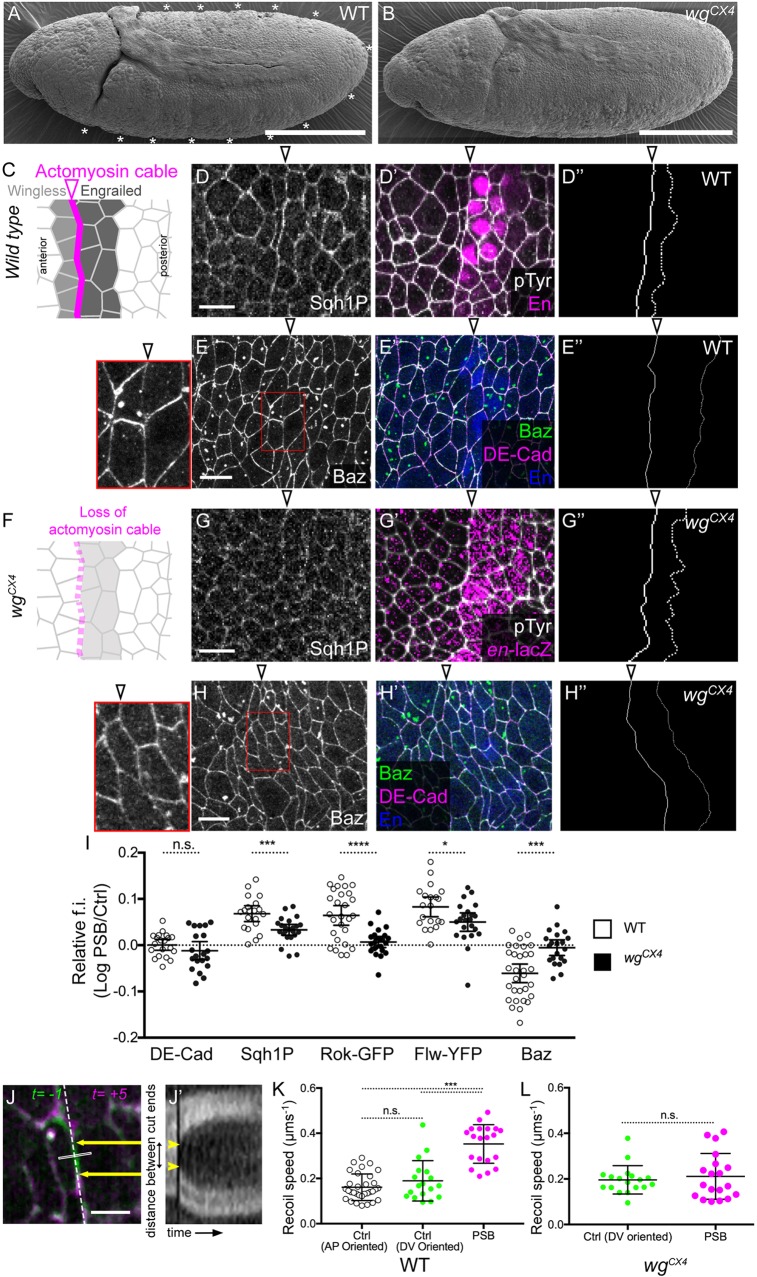
**Planar polarities and actomyosin contractility at PSBs in wild-type and *wingless* mutant embryos.** (A,B) Representative wild-type (WT) (*n*=30) (A) and *wg^CX4^* (*n*=22) (B) stage 10 embryos imaged by scanning electron microscopy. Scale bars: 100 µm. Parasegmental grooves (asterisks) apparent in WT embryos are absent in *wg^CX4^* embryos. (C,F) PSBs, corresponding to the interfaces between *wingless-* and *engrailed*-expressing cells, enrich actomyosin in WT embryos (C). This enrichment is mostly lost in *wg^CX4^* embryos (F). (D,E,G,H) Immunostaining against Sqh1P or Baz in WT or *wg^CX4^* embryos (close-ups shown for Baz). Scale bars: 10 µm. (D′,E′, G′,H′) Corresponding merges with an AJ marker, E-Cadherin (DE-Cad) or phosphotyrosine (pTyr), and PSB marker, Engrailed (En) or *en-lacZ* (*en-lacZ* is used to identify PSBs in *wg* mutants; see Materials and Methods). (D″,E″,G″,H″) Tracings of the PSB (solid line) and control cell-cell contacts (dotted line) to quantify the signal at the level of AJs. (I) Quantification of the fluorescence intensities (f.i.) of proteins along PSBs relative to control interfaces, in WT and *wg^CX4^* embryos, as log_10_ (DE-Cad in WT, *n*=20 PSBs, in *wg^CX4^*, *n*=20; Sqh1P in WT, *n*=20, in *wg^CX4^*, *n*=23; Rok-GFP in WT, *n*=26, in *wg^CX4^*, *n*=23; Flw-YFP in WT, *n*=20, in *wg^CX4^*, *n*=22; Baz in WT, *n*=31, in *wg^CX4^*, *n*=21). Error bars show mean±95% confidence interval (CI). Comparisons between WT and *wg^CX4^* from Student's *t*-tests: DE-Cad, *P*=0.256 (n.s.); Sqh1P, ***P=0.0008; Rok-GFP, ****P<0.0001; Flw-YFP, *P=0.0237; Baz, ***P=0.0002. (J-L) Laser ablations to probe junctional tension at PSBs. (J) Overlay before (green) and after (magenta) ablation of a single cell-cell junction at the PSB (the white rectangle indicates the ablation zone). Scale bar: 5 µm. (J′) Kymograph of the signal along the dashed line in J used to measure distance between cut ends over time (arrows in J; arrowheads in J′). (K,L) Recoil speed upon ablation of cell-cell junctions in WT (K) and *wg^CX4^* embryos (L) at PSBs or control junctions parallel to AP or DV axes. Control AP junctions in WT, *n*=32 ablations; control DV junctions in WT, *n*=18; PSBs in WT, *n*=20; control DV junctions in *wg^CX4^*, *n*=18; PSBs in *wg^CX4^*, *n*=19. Error bars show mean±s.d. Comparisons in K from a Kruskal–Wallis test: AP control versus DV control, *P*=0.780 (n.s.); PSB versus control, ***P<0.0001. Comparison in L from a Mann–Whitney test: *P*=0.910 (n.s.). In all figures, anterior is left and dorsal up, unless otherwise stated. Open arrowheads label PSBs.

The transient parasegmental grooves that form from mid-stage 10 and throughout stage 11 at the site of the compartmental boundaries between Wingless- and Engrailed-expressing cells (PSBs) are absent in *wingless* mutants (Larsen et al., 2008) (Fig. 1A,B). To understand what could control epithelial folding at PSBs downstream of Wingless signalling, we analysed the cortical enrichment of proteins known to be planar polarized earlier in development during germ-band extension (stages 7-8) at dorsoventral (DV)-oriented cell-cell junctions, which include early PSBs. For example, F-actin, Myosin II and Rho kinase (Rok) are enriched, whereas Bazooka (Baz, Par-3 homologue) and E-Cadherin are depleted, at DV-oriented junctions during germ-band extension (Bertet et al., 2004; Blankenship et al., 2006; Farrell et al., 2017; Levayer et al., 2011; Simões et al., 2010; Tetley et al., 2016; Zallen and Wieschaus, 2004). We had shown previously that F-actin and two reporters for nonmuscle Myosin II, Zip-GFP (*zip* encodes MHC) and Sqh-GFP (*sqh* encodes MRLC), are enriched at PSBs at germ-band extended stages (stages 9 to 11) (Monier et al., 2010). Here, we developed a method to quantify the enrichment or depletion of proteins at the level of AJs along the PSBs, relative to control columns of DV-oriented junctions (see Materials and Methods). We confirm that Myosin II is not only enriched but also activated at the PSBs at germ-band extended stages: the mono-phosphorylated form of MRLC [recognized by the Sqh1P antibody (Zhang and Ward, 2011)] accumulates at the PSB (Fig. 1C-D″,I). Moreover, we find that Rok (using Rok-GFP) is enriched at the PSB (Fig. S1B-B″), whereas Baz is depleted (Fig. 1E-E″,I). Therefore, the key planar polarities established during germ-band extension are maintained at germ-band-extended stages specifically and robustly at the PSBs.

We find, however, one notable difference in our survey of polarities: whereas E-Cadherin levels are depleted at DV junctions in germ-band extension (Blankenship et al., 2006; Levayer et al., 2011), this does not appear to be the case at PSBs at stage 10, where levels are the same as in other junctions (Fig. 1I, Fig. S1A-A″). This might be linked to the distinct behaviours of cells during axis extension versus boundary formation; when cells intercalate during germ-band extension, contacts need to be remodelled and the depletion of E-Cadherin might facilitate this (Warrington et al., 2013). In contrast, later in development, the PSB is a stable interface between neighbouring cell populations, where normal adhesion might be required for boundary function.

Planar polarities during germ-band extension are under the control of the pair-rule genes (Bertet et al., 2004; Paré et al., 2014; Zallen and Wieschaus, 2004). We have shown recently that PSB interfaces enrich actomyosin more than other DV-oriented interfaces during germ-band extension, and this is also likely to require pair-rule input, because this enrichment is not disrupted in *wingless* mutants (Tetley et al., 2016). In contrast, later on, the maintenance of robust actomyosin polarization at PSBs at germ-band extended stage does require Wingless signalling (Monier et al., 2010). Confirming this, the enrichment of Sqh1P is significantly decreased at PSBs in *wingless* null mutants (Fig. 1F-G″,I). Moreover, the enrichment of Rok and depletion of Baz at PSBs is lost (Fig. 1I). Therefore, the maintenance of key planar polarities at the PSBs at germ-band extended stages requires Wingless signalling.

### Actomyosin enrichments at PSBs correlate with higher interfacial tension at boundary cell-cell contacts

An actomyosin enrichment along the compartmental boundary interfaces suggests an increase in cortical tension there, which could be important for fold formation. Previously, we had provided evidence of higher tension along the PSBs at germ-band extended stages, by showing that the PSBs are straighter than control DV-oriented columns of interfaces, and that this increased straightness is lost in *wingless* mutants (Monier et al., 2010). Here, we probe more directly junctional tension at the PSBs, using laser ablation to measure the speed of junctional recoil as previously (Tetley et al., 2016). We find that, at stage 10, the recoil velocities are on average about twice as fast at PSBs compared with nonboundary DV- or AP-oriented junctions (Fig. 1J-K). Controls show that the PSB junctions used for ablation are enriched in Myosin II compared with other DV-oriented junctions as expected and their lengths are comparable, ruling out an effect of junction size (Fig. S1D,D′).

We repeated the ablations at PSB junctions in a *wingless* mutant, and show that the increase in actomyosin-dependent tension is lost (Fig. 1L, ablation controls in Fig. S1E,E′). Note that PSBs can be located in *wingless* mutants because of a weak remaining enrichment in actomyosin at the boundary (quantified in Fig. 1I and Fig. S1E) (see also Fig. 2M in Tetley et al., 2016). This weak enrichment is not sufficient to maintain a high tension at the boundary, because both our laser ablation experiments (this study) and quantifications of boundary straightness (Monier et al., 2010) demonstrate that tension at PSB boundaries and control interfaces become identical in *wg^CX4^* mutants. This confirms that Wingless signalling is required for increasing actomyosin-dependent junctional tension specifically at the PSBs. Because parasegmental groove formation also requires Wingless signalling (Larsen et al., 2008) (Fig. 1B), this result suggests that epithelial folding at PSBs is a consequence of increased junctional actomyosin tension.

### Hyperactivation of Myosin II via knockdown of the Myosin II phosphatase Flw increases epithelial folding at PSBs

Although the above results suggest that boundary actomyosin enrichments are required for parasegmental groove formation, they do not appear sufficient*.* Indeed, we have recently shown that actomyosin enrichments at PSBs are detectable as early as 20 min after the beginning of germ-band extension (stage 8), and that the PSB interfaces are already under higher interfacial tension than other interfaces, as shown by measures of straightness and laser ablation experiments (Tetley et al., 2016). Therefore, the PSBs are continuously enriched in actomyosin from stage 8 onwards (Monier et al., 2010, 2011; Tetley et al., 2016). However, the parasegment grooves appear only around mid-stage 10, ∼2 h later (Martinez-Arias, 1993). This suggests that ‘brakes’ might exist that limit folding at boundary actomyosin enrichments. A possible ‘brake’ is Flapwing (Flw), a component of the main phosphatase that negatively regulates nonmuscle Myosin II activity (Vereshchagina et al., 2004). Screening the Cambridge Protein Trap Insertion (CPTI) collection (Lowe et al., 2014; Lye et al., 2014), we have identified Flw-YFP as one of several proteins enriched at PSBs (Naylor, 2011). Here, we show that Flw-YFP is enriched at the PSBs at germ-band extended stages and that this enrichment is significantly reduced in *wingless* null mutants (Fig. 1I, Fig. S1C-C″). We also find that Flw-YFP systematically co-localizes with activated Myosin II (Sqh1P) in early embryos, such as at the cellularization front, the ventral furrow and at DV-oriented cell-cell contacts during germ-band extension (Fig. S2A-C″). Movie 1 also shows that Flw-YFP is dynamically present in medial flows as Myosin II (Rauzi et al., 2010). This suggests an important role of Flw in regulating Myosin II activity at morphogenetic sites.

**Fig. 2.**
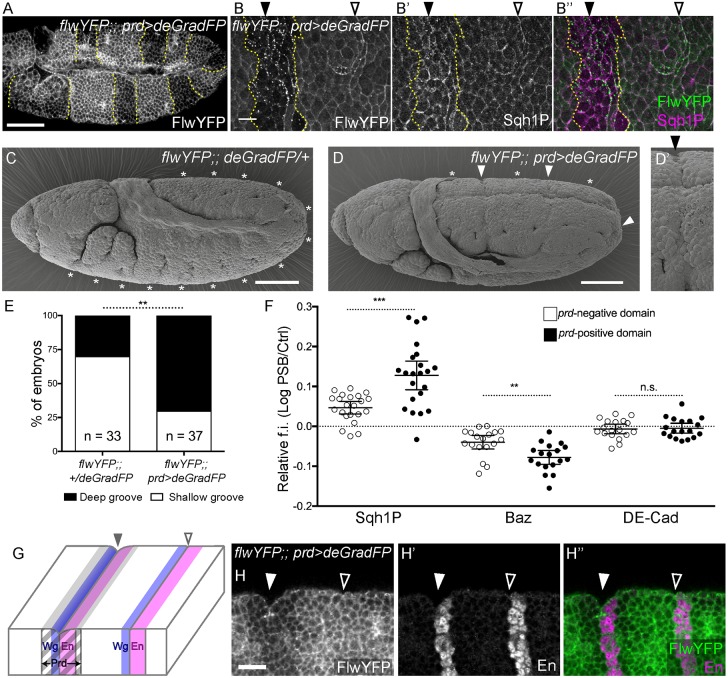
**PSB grooves are deepened by depletion of the Myosin-II phosphatase Flapwing.** (A) Immunostaining against GFP reveals degradation of Flw-YFP in an embryo expressing *UAS-deGradFP* in the *prd-Gal4* domain (yellow dotted lines). Scale bar: 50 µm. (B-B″) The same genotype at higher magnification, immunostained against GFP (B), Sqh1P (B′) and merged (B″). Scale bar: 10 µm. Filled arrowheads indicate PSBs in *prd-Gal4* domains; open arrowheads indicate control PSBs. (C-D′) SEM images of late stage 10 control and deGradFP embryos. Asterisks indicate shallow parasegmental grooves in control embryos; filled arrowheads indicate deepened parasegmental grooves in Flw-depleted domains (close-up in D′). Scale bars: 50 µm. (E) Blind quantification of embryos with shallow only versus deep grooves in sibling embryos shown in C and D. Comparison from Fisher's exact test, **P=0.0016. (F) Quantification of the fluorescence intensities (f.i.) of proteins at PSBs in *deGradFP*-expressing and -nonexpressing domains (*prd-Gal4* positive or negative), relative to control interfaces, as log_10_ (for both domains, Sqh1P, *n*=22 PSBs; Baz and DE-Cad, *n*=18). Error bars show mean±95% CI. Comparisons between *prd-Gal4*-positive and -negative PSBs from Mann–Whitney tests: Sqh1P, ***P=0.0002; Baz, **P=0.0016; DE-Cad, *P*=0.9626 (n.s.). (G) PSB position and fold depth relative to Wg, En and *prd-Gal4* expression domains. (H-H″) Immunostaining against GFP (H) and En (H′) (H″, merge) showing the deep groove at the PSB in the Flw-depleted domain (filled arrowheads) and the shallow groove (open arrowheads) in the control domain. Scale bar: 20 µm.

To disrupt this putative negative regulation, we used the deGradFP system to degrade YFP/GFP-tagged proteins (Caussinus et al., 2012). Because YFP is inserted at the *flw* locus and the resulting Flw-YFP insertion is homozygous viable, all molecules of Flw are susceptible to be degraded. When nanobodies against YFP (*UAS-deGradFP*) are expressed under the control of *paired-Gal4* (*prd-Gal4*), Flw-YFP is efficiently depleted in *prd-Gal4*-expressing domains (Fig. 2A). Although Flw-YFP is normally cortical (Fig. S2A-C″), it is lost from the membranes in the *prd-Gal4* domains and accumulates as bright dots in the cytoplasm (Fig. 2B). We find that Sqh1P levels are elevated in *prd-Gal4* domains (Fig. 2B-B″), indicating that Flw depletion results in Myosin II hyperphosphorylation. We also detect a higher enrichment in Sqh1P along the PSBs located in *prd-Gal4* domains (Fig. 2F). This is consistent with the key reported phenotype of *flw* mutants being an increase in Sqh phosphorylation (Sun et al., 2011; Vereshchagina et al., 2004). To check whether Flw could have other effects, we also quantified some of the planar polarities examined previously. We found that Baz remains depleted at the PSBs in *prd-Gal4* regions (with some increase in the level of depletion) and E-Cadherin is unchanged (Fig. 2F). Because there is a reported effect of Flw on Moesin and Merlin phosphorylation (Yamamoto et al., 2013; Yang et al., 2012), we also quantified phospho-Moesin (pMoe) at the PSBs. We found that pMoe is enriched there, but the level of enrichment did not change in Flw-depleted *prd-Gal4* domains, ruling out this target (Fig. S2D).

Remarkably, deGraFP-mediated Flw depletion is associated with a specific morphogenetic phenotype: the normally shallow parasegmental grooves (Fig. 2C) are now deep in each *prd-Gal4* domain (Fig. 2D-E,G-H″). This effect is specific to the PSBs: no other epithelial folds appear in the domains depleted for Flw. Together, these results show that Flw negatively regulates fold formation at PSBs, most likely through direct inhibition of Myosin II activity. This indicates that fold formation at PSBs is normally suppressed in wild-type embryos.

### Ectopic PSBs are associated with deeper folds compared with endogenous PSBs

**Fig. 3.**
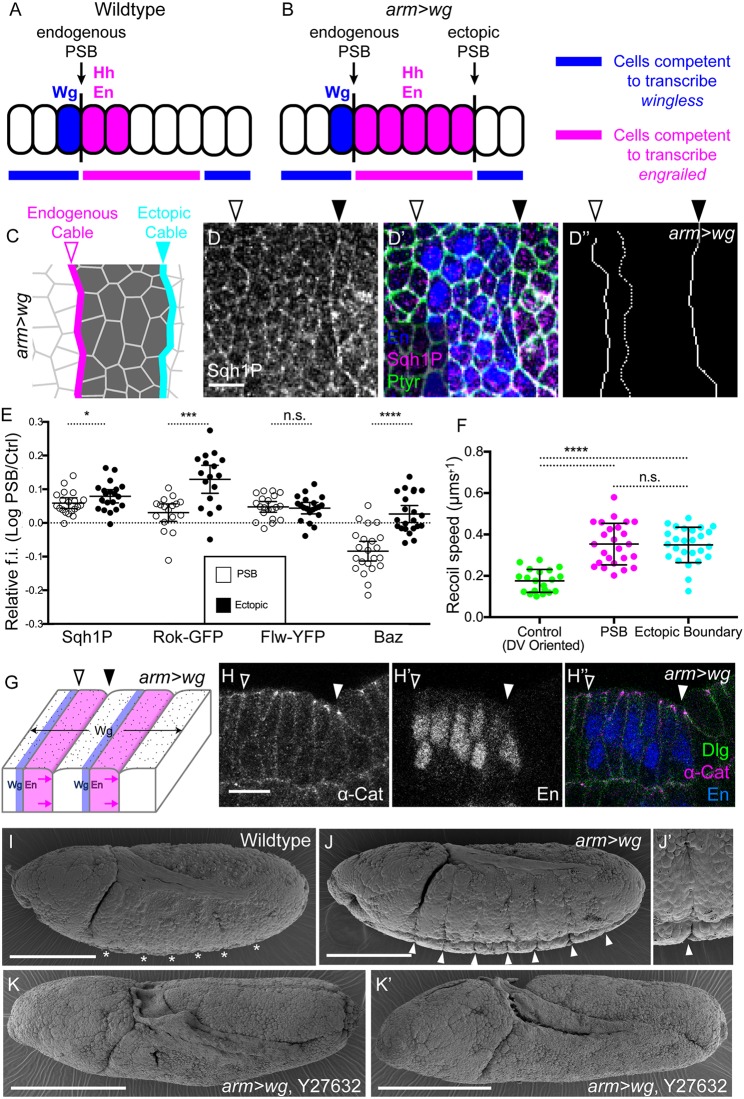
**Increased epithelial folding at ectopic PSBs in *wingless-*overexpressing embryos.** (A,B) Location of *wg-*, *hh-* and *en*-expressing cells relative to endogenous and ectopic PSBs at stage 10. Ectopic PSBs form at posterior edges of the enlarged Engrailed domain in *arm>wg* embryos. (C) Position of the actomyosin enrichment at endogenous (magenta) and ectopic (cyan) PSBs in *arm>wg* embryos. (D,D′) Immunostaining of *arm>wg* early stage 10 embryos against Sqh1P (D), En and pTyr (merged in D′). Scale bar: 10 µm. (D″) Traces of endogenous and ectopic PSBs (solid lines) and control junctions (dotted line). (E) Quantification of the fluorescence intensities (f.i.) of proteins in *arm>wg* embryos along the endogenous (open circles) and ectopic (solid circles) PSB junctions, relative to control interfaces, as log_10_ (for both boundaries, Sqh1P, *n*=20; Rok-GFP, *n*=17; Flw-YFP, *n*=20; Baz, *n*=22). Error bars show mean±95% CI. Comparisons between PSBs and ectopic boundaries from Student's *t*-tests: Sqh1P, *P=0.025; Rok-GFP, ***P=0.0002; Flw-YFP, *P*=0.76 (n.s.); Baz, ****P<0.0001. (F) Recoil speeds following laser ablation of endogenous and ectopic PSB cell junctions, and control DV-oriented junctions. Control DV junctions, *n*=20 ablations; PSB, *n*=25; ectopic, *n*=26. Error bars show mean±s.d. Comparisons from one-way ANOVA: DV controls versus PSBs or ectopics, ****P<0.0001; PSB versus ectopics, *P*=0.998 (n.s.). (G) Position of the shallow and deep folds at endogenous and ectopic PSBs, respectively, in *arm>wg* embryos. (H-H″) Sagittal view showing difference in folding at the endogenous and ectopic PSBs of an *arm>wg* embryo stained for alpha-Catenin (α-Cat) (H), En (H′) and merged with Discs Large (Dlg) (H″). Scale bar: 10 µm. (I-J′) SEM of stage 10 (I) wild-type and (J,J′) *arm>wg* embryos. Endogenous PSBs barely indent the surface of the embryo (asterisks in I), whereas ectopic PSBs form deep grooves (J, close-up in J′). WT, *n*=30 embryos; *arm>wg*, *n*=62 embryos, of which 56 had deep folds. (K,K′) SEM of stage 10 *arm>wg* embryos injected with Rho kinase inhibitor Y-27632, showing two examples; *n*=10 embryos, of which 9 had no grooves. Scale bars: 100 µm. Open arrowheads indicate endogenous PSBs; filled arrowheads indicate ectopic PSBs.

To further investigate the link between actomyosin contractility and epithelial folding, we generated ectopic PSBs by expressing *wingless* (*wg*) in the whole epithelium (*arm-Gal4/UAS-wg*, hereafter *arm>wg*) (Larsen et al., 2008; Sanson et al., 1999). In wild-type embryos, Wg signals at short range from the cells anterior to the PSB, to maintain *engrailed* (*en*) transcription in the cells posterior to the PSB (Vincent and Lawrence, 1994) (Fig. 3A). In *arm>wg* embryos, ectopic Wg maintains *en* expression in a larger domain spanning approximately half the parasegment, which corresponds to the cells competent to transcribe *en* (Fig. 3B). The posterior margin of this enlarged domain now abuts the other half of the parasegment, where cells are competent to transcribe *wg*. This new interface between the enlarged En domain (which also expresses *hedgehog*, *hh*) and the Wg-transcription-competent cells can be viewed as an ectopic PSB, because it replicates the transcriptional and signalling environment of the endogenous PSB. Consistent with this notion, we find that Sqh1P is enriched at the cell-cell contacts of these ectopic PSBs relative to control interfaces, similarly to the endogenous PSBs present in the same embryos (Fig. 3C-E). The positive and negative regulators of Myosin II, Rok and Flw, are also enriched at ectopic PSBs (Fig. 3E). Furthermore, laser ablations of cell-cell contacts at ectopic PSBs show that junctional tension is elevated there as for endogenous PSBs (Fig. 3F, Fig. S3B,B′). We conclude that ectopic PSBs recapitulate the highly contractile actomyosin interfaces of endogenous PSBs.

Ectopic boundaries are associated with an epithelial fold (Larsen et al., 2008), providing additional evidence that actomyosin enrichment at PSBs promotes epithelial folding. There is, however, a key difference: folds at ectopic PSBs are deep when compared with endogenous parasegmental grooves (Fig. 3G-J′). The folding appears even more pronounced than what is observed when actomyosin contractility is elevated in the Flw depletion (compare Fig. 3J′ and Fig. 2D′). The deep folds require actomyosin contractility because they are absent (as well as the endogenous grooves) in embryos injected with the Rok inhibitor Y-27632 (Fig. 3K,K′). However, we cannot find evidence of a further increase in junctional actomyosin contractility compared with the endogenous PSBs that could explain the deep folds: enrichments of Sqh1P at ectopic and endogenous PSBs are similar (Fig. 3E) and the recoil speeds upon laser ablation are indistinguishable (Fig. 3F).

To test whether ectopic PSBs were systematically associated with deeper folds, we examined two other genotypes, embryos expressing *rho-Gal4/UAS-wg* (*rho>wg*) and null mutants for the gene *naked cuticle* (*nkd*). *Rho-Gal4* is expressed in a ventral stripe a few cell diameters wide on either side of the ventral midline (Ip et al., 1992). When *wingless* is ectopically expressed using this driver, the En domain is enlarged in the corresponding ventral region and a ventral, stubby, ectopic PSB forms in each parasegment, which is enriched in actomyosin (Fig. S3C,E-E″). A deep fold forms that does not extend beyond the extremity of the short ectopic PSBs, suggesting that the folding might be cell autonomous (Fig. S3D,D′). In a *nkd* null embryo, Wingless signalling is altered and signals more weakly, but over a longer distance, resulting in an enlarged En domain and an ectopic PSB (Martinez Arias et al., 1988; Zeng et al., 2000). These ectopic PSBs enrich actomyosin and produce a deep fold (Fig. S3F-H″). Thus, ectopic PSBs produced by different genetic manipulations all enrich actomyosin at their interfaces like the endogenous PSBs. However, unlike endogenous PSBs, they are systematically associated with deep folds rather than shallow ones.

What could explain the difference in degree of folding between endogenous and ectopic PSBs? We have proposed above that ‘brakes’ exist that suppress folding at actomyosin-enriched boundaries and shown that Flw is one of these brakes (Fig. 2). Perhaps not all of the brakes are recapitulated at ectopic boundaries, so we examined the planar polarization of factors we quantified earlier for endogenous boundaries (Fig. 3E). Flw-YFP and Sqh1P are enriched at the same level in both endogenous and ectopic boundaries, which rules out a role for Flapwing. We find, however, clear differences for two other factors: Rok is more enriched at ectopic boundaries, while Baz is no longer depleted (Fig. 3E, Fig. S3A-A″). Because Sqh1P enrichment (also Sqh-GFP enrichment, Fig. S3B) and junctional tension are indistinguishable at endogenous versus ectopic PSBs (Fig. 3E,F), the increase in Rok does not appear to increase junctional actomyosin contractility at the ectopic PSBs. However, it could affect folding through other pathways or modify lateral, rather than junctional contractility (see Discussion). The absence of depletion of Baz at ectopic boundaries was intriguing because Baz has been implicated in the initiation of dorsal folds in the early embryo (Wang et al., 2012). This prompted us to analyse further a putative role of Baz in controlling epithelial fold depth at PSBs.

### Bazooka increases the depth of epithelial folding at PSBs

**Fig. 4.**
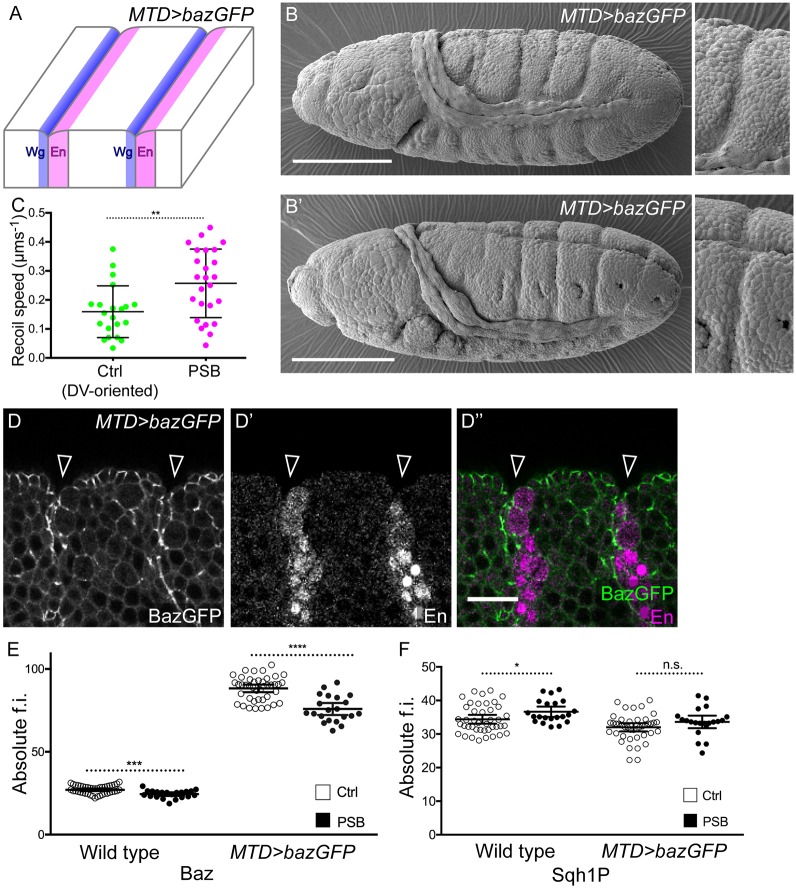
**Baz overexpression increases epithelial folding at actomyosin-enriched boundaries.** (A) Positions of deep folds at endogenous PSBs in Baz-overexpressing embryos (*MTD>bazGFP*). (B,B′) SEM of *MTD>bazGFP* embryos (B) at stage 10 and (B′) stage 11, showing deep folding at PSBs. *n*=28 embryos (24 show deepened folds, 85.7%). Scale bars: 100 µm. (C) Recoil speeds following laser ablation of DV-oriented control and PSB junctions in *MTD>bazGFP* embryos. Control DV junctions, *n*=21 ablations; PSB, *n*=25. Error bars show mean±s.d. Comparison from a Student's *t*-test: **P=0.0032. (D-D″) Grazing section of an early stage 10 *MTD>bazGFP* embryo, immunostained against GFP (D) and Engrailed (D′) (merged in D″), showing deep PSB folds. Scale bar: 20 µm. (E,F) Quantification of the absolute fluorescence intensities (f.i.) of Baz (E) and Sqh1P (F) at PSBs and control DV-oriented interfaces in wild-type and *MTD>bazGFP* embryos. For PSBs (in both WT and *MTD>bazGFP* embryos, for both Sqh1P and Baz), *n*=21 boundaries; controls, *n*=42. Error bars show mean±95% CI. Comparisons from Student's *t*-tests: Baz in WT, ***P=0.0001; Baz in *MTD>bazGFP*, ****P<0.0001; Sqh1P in WT, *P=0.0368; Sqh1P in *MTD>bazGFP*, *P*=0.139 (n.s.).

To test whether Baz could have an impact on epithelial folding at PSBs, we overexpressed UAS-Baz-GFP in the embryo using maternal Gal4 drivers (Maternal triple driver, MTD; *MTD>bazGFP*). We find that Baz overexpression causes the formation of deep folds specifically at the PSBs and nowhere else (Fig. 4A-B′). These folds are much deeper than wild-type parasegmental grooves. As for ectopic PSBs in *arm>wg* embryos, this effect cannot be explained by an increase in junctional actomyosin contractility. First, laser ablations of PSB versus control DV-oriented junctions give similar recoil velocities (compare Fig. 4C with Fig. 1K or Fig. 3F), with a ratio of ∼2 between PSBs and control junctions (Fig. 4C, controls in Fig. S4B,B′). Second, the absolute quantities of Sqh1P are equivalent between Baz-overexpressing embryos and wild type, for both PSBs and DV-oriented control junctions (Fig. 4F). Third, Sqh1P is similarly enriched at PSBs in both genotypes (Fig. S4A). So in terms of junctional actomyosin enrichment and tension, the PSBs in Baz-overexpressing embryos are indistinguishable from those in wild-type embryos. Note that Baz is still found depleted at PSBs relative to control junctions in Baz-overexpressing embryos (Fig. S4A), suggesting that the signals controlling its depletion at boundaries are functioning normally. As expected, however, the absolute levels of Baz are much higher in Baz-overexpressing embryos (Fig. 4E), indicating that it is the overall increase in Baz that promotes deep epithelial folding at actomyosin-enriched boundaries.

**Fig. 5.**
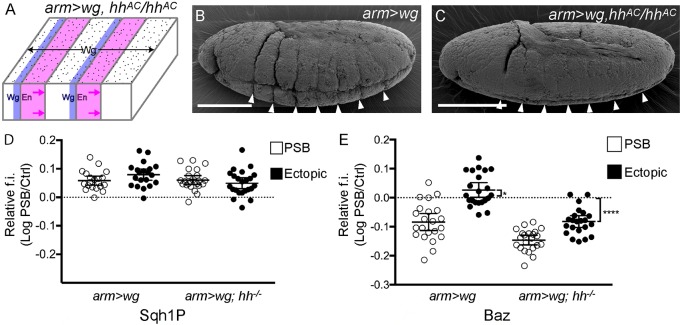
**Fold suppression and Baz depletion is rescued at ectopic PSBs in absence of Hedgehog.** (A) Lack of deep grooves at endogenous and ectopic PSBs, in *arm>wg* embryos in a null *hh* mutant background. (B,C) SEM of stage 10 embryos with *arm>wg* (B) showing the deep grooves at ectopic PSBs (*n*=62 embryos), which are much shallower in *arm>wg, hh^AC^/hh^AC^* (C) (*n*=19 embryos, of which 17 had shallow grooves). Scale bars: 100 µm. (D,E) Quantification of the fluorescence intensities (f.i.) of Sqh1P (D) and Baz (E) in *arm>wg* and *arm>wg, hh^AC^/hh^AC^* embryos along the PSB (open circles) and ectopic (filled circles) junctions, relative to control cell interfaces, as log_10_. Error bars show mean±95% CI. Sqh1P in *arm>wg*, *n*=20; Sqh1P in *arm>wg, hh^AC^/hh^AC^*, *n*=23; Baz in *arm>wg*, *n*=22; Baz in *arm>wg, hh^AC^/hh^AC^*, *n*=22. Comparisons in E from one-sample Student's *t*-tests: Baz at ectopics in *arm>wg*, difference from 0, *P=0.0399; Baz at ectopics in *arm>wg, hh^AC^/hh^AC^*, difference from 0, ****P<0.0001.

To test this further, we searched for experimental conditions that could rescue deep epithelial folding. We show above that Wingless signalling is required for Baz depletion at the endogenous PSBs (Fig. 1), but that Baz is not depleted at ectopic boundaries in *arm>wg* embryos (Fig. 3). So, a possibility could be that a signal is inhibiting Wingless-dependent depletion of Baz at ectopic boundaries. A likely signal is Hedgehog (Hh) (Hatini and DiNardo, 2001; Sanson, 2001); it has been found to antagonize the regulation of specific genes by Wingless in the region posterior to the Engrailed domain (Sanson et al., 1999) and to increase the lysosomal degradation of Wingless in this region (Dubois et al., 2001). To test whether Hedgehog signalling could have an opposite effect to Wingless signalling on Baz levels, we quantified Baz at ectopic boundaries in *arm>wg* embryos in a null mutant background for *hedgehog* (*arm>wg [hh^−/−^]*)*.* Strikingly in these embryos, fold depth at ectopic boundaries is reduced and now similar to endogenous boundaries (Fig. 5A-C). Moreover, we find that Baz depletion at ectopic boundaries in *arm>wg [hh^−/−^]* is now indistinguishable from the depletion of Baz at endogenous PSBs in *arm>wg* (Fig. 5E) or wild-type (Fig. 1I) embryos. This provides additional evidence that Wingless depletes Baz levels at endogenous PSBs and, when Hh signalling is removed, at ectopic PSBs. Interestingly, the depletion of Baz at endogenous boundaries in *arm>wg [hh^−/−^]* embryos is also enhanced (Fig. 5E), so Hedgehog signalling antagonizes Wingless-dependent Baz depletion at both endogenous and ectopic PSBs. Thus Wingless and Hedgehog signalling have opposite effects on Baz levels at PSBs, endogenous and ectopic. Consistent with findings from Baz overexpression embryos (Fig. 4F), we do not find a difference in Sqh1P levels in *arm>wg* and *arm>wg [hh^−/−^]* embryos, for either endogenous or ectopic PSBs (Fig. 5D), indicating that the effect of Baz on folding is independent of junctional actomyosin contractility.

The above results are consistent with a role of Baz in promoting folding, because its presence at boundaries correlates with folding depth. To test for sufficiency, we attempted to abolish folding by knocking down Baz. We used the deGradFP system again, this time to deplete Baz-GFP levels in an *arm>wg* background, to ask whether this would suppress deep folding at ectopic PSBs. We demonstrate first that deGradFP depletion of Baz-GFP is effective (Fig. S4C). We then depleted Baz levels in embryos which are either homozygous for Baz-GFP or transheterozygous for Baz-GFP and a null allele of *baz* (*baz^XR11^*), to test two different levels of depletion. In both cases, the epithelium begins to depolarize (Fig. S4D,E), consistent with the known phenotype of *baz* mutants (Muller and Wieschaus, 1996). However, Baz depletion in these embryos is not sufficient to abolish the deep folds at ectopic PSBs. So although Myosin II inhibition does suppress folding at both endogenous and ectopic PSBs (Fig. 3K,K′), Baz depletion does not. This suggests that either our removal of Baz is not early enough in development to inhibit folding or that other factors in addition to Baz promote fold formation at PSBs.

### Epithelial folding at PSBs is independent of apical constriction or AJ lowering

**Fig. 6.**
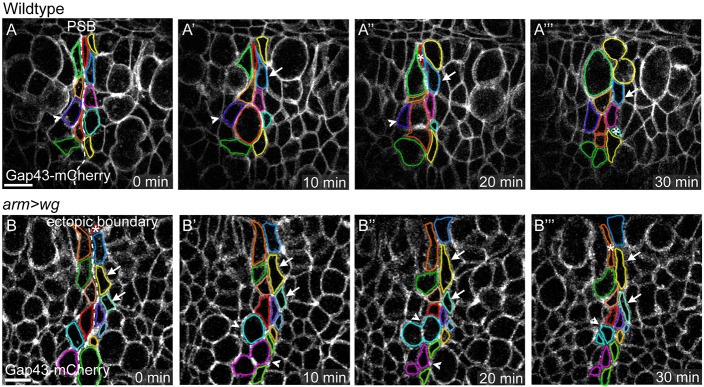
**Cell behaviours during folding at endogenous and ectopic PSBs in live embryos.** (A-B‴) Frames at 10-min intervals from time-lapse imaging of a live stage 10 embryo expressing Gap43-mCherry, with cells abutting the PSB highlighted, in a wild-type (A-A‴) and an *arm>wg* (B-B‴) embryo. Dashed lines highlight the boundaries; the asterisks mark cells that delaminate from the epithelium; arrows indicate cells undergoing intercalation; arrowheads indicate cells undergoing intercalation events associated with cell divisions. Anterior is left and ventral is up. Scale bars: 10 µm.

To understand better the mechanisms leading to folding at PSBs, we examined cell behaviours during both endogenous and ectopic fold formation in live embryos, for a period of 30 min (Fig. 6). We used Gap43-Cherry to image the cell membrane and identified PSBs either by the presence of an actomyosin enrichment (reported by Sqh-GFP) for wild-type embryos (Fig. 6A) or by expression of Eve-GFP (which weakly labels cells immediately posterior to the PSBs at this stage) for *arm>wg* embryos (Fig. 6B). During extended germ-band stages (9 to 11), the epidermal cells are dividing frequently (Martinez-Arias, 1993), and in the movie frames shown in Fig. 6, several cells contacting either the endogenous or the ectopic PSBs are dividing. Consistent with our previous finding that the PSB acts as a mechanical barrier (Monier et al., 2010), the resulting daughter cells do not cross the PSBs. However, often the daughter cells move along the PSBs, intercalating between neighbours (Fig. 6, arrowheads). Nondividing boundary cells are also sometimes intercalating (Fig. 6, arrows). Other boundary cells delaminate (Fig. 6, asterisks), having acquired a very elongated apical shape along the boundary. These behaviours suggest that boundary cells are being forced to exchange neighbours, elongate and sometimes delaminate on either side of the boundary, as a consequence of mechanical tension along the boundary.

**Fig. 7.**
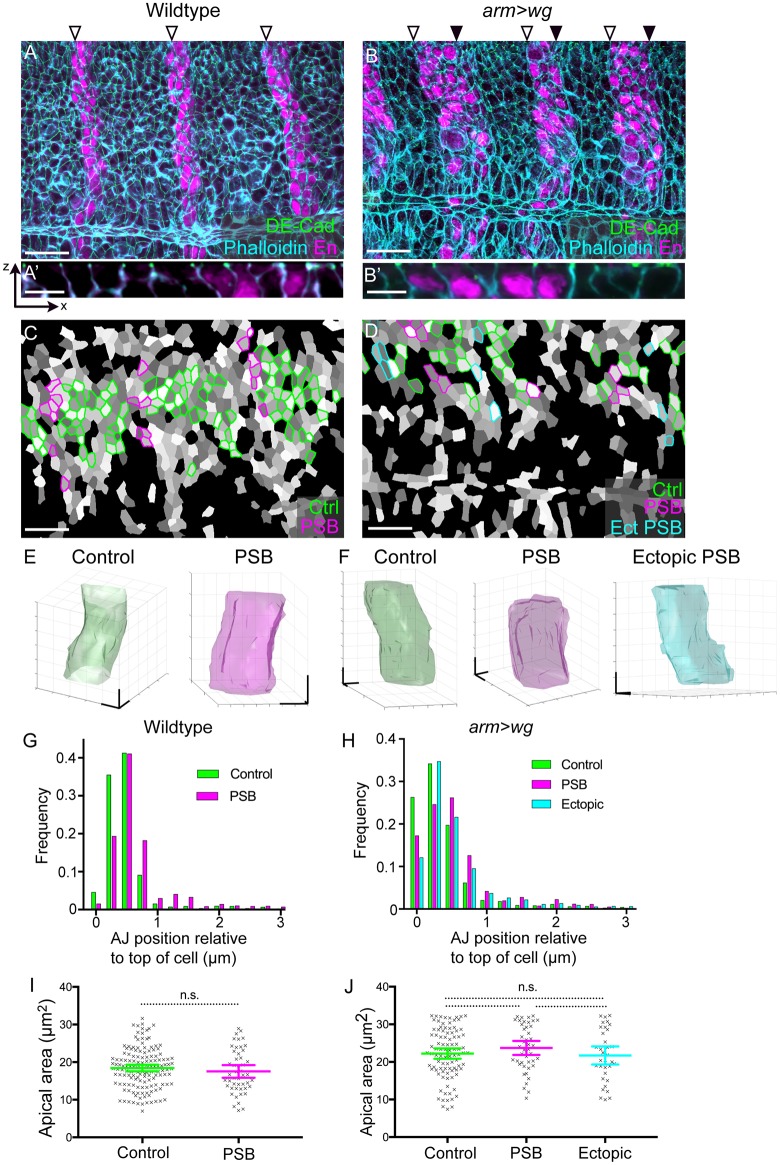
**Measuring AJ lowering and apical constriction at endogenous and ectopic PSBs.** (A,B) Confocal stack projections of stage 10 wild-type and *arm>wg* embryos immunostained for E-Cadherin (green), phalloidin (cyan) and Engrailed (magenta). The positions of endogenous (open arrowheads) and ectopic (filled arrowheads) PSBs are indicated. Scale bars: 20 µm. (A′,B′) Close-up of *x*-*z* optical sections through the stacks shown in A and B. Scale bars: 5 µm. (C,D) Cell segmentation of image stacks shown in A and B. Cells depicted in green, magenta and cyan are control cells and endogenous and ectopic PSB-abutting cells, respectively. Scale bars: 20 µm. (E,F) 3D cell reconstructions of representative example cells from wild-type (E) and *arm>wg* (F) embryos. Control cells, green; endogenous PSB-abutting cells, magenta; ectopic PSB-abutting cell, cyan. Scale bars: 2 µm. (G,H) Histograms showing the distance separating the AJs from the top of the cell for control, endogenous PSB and ectopic PSB cell-cell junctions, in wild-type (G) and *arm>wg* (H) embryos. In wild-type embryos, the mean AJ positions are 0.67 µm (below the top of the cell) for controls and 0.86 µm for PSBs (*n*=2120 pixels in PSB junctions; *n*=2481 in controls). In *arm>wg* embryos, the mean AJ positions are 0.71 µm for controls, 0.84 µm for endogenous PSBs and 1.06 µm for ectopic PSBs (*n*=1139 pixels in endogenous PSB junctions; *n*=1309 in controls; *n*=1398 in ectopic PSBs). Outliers (>3 µm between AJ and top of cell) are omitted; they account for 3.1% and 2.5% of the data in wild-type and *arm>wg* embryos, respectively. (I,J) Quantification of apical areas of control cells and cells abutting the endogenous and ectopic PSBs, in wild-type (I) and *arm>wg* (J) embryos (cell numbers: in wild-type controls, *n*=144; PSBs, *n*=48; in *arm>wg* controls, *n*=94; PSBs, *n*=44 PSBs; ectopic PSBs, *n*=34). Comparison in I from a Student's *t*-test: *P*=0.338 (n.s.). Comparisons in J from a Kruskal–Wallis test: all pairs, n.s. Error bars show mean±95% CI. Three embryos of each genotype (shown in Fig. S5) were analysed in G-J.

These movies, however, did not reveal any obvious, stereotypical cell behaviour that could explain fold formation. Known mechanisms for fold formation include apical constriction (Martin and Goldstein, 2014) and AJ lowering (Wang et al., 2012). In order to use a quantitative approach to search for such mechanisms, we analysed fixed embryos immediately before or just at the beginning of fold formation, imaging the whole cell volume by marking actin using fluorescently labelled phalloidin in WT and *arm>wg* embryos (Fig. 7A-B′). We then segmented the 3D volumes of example cells either abutting PSBs or not (Fig. 7C-F), and measured the position of the AJs relative to the apical top of the cells (see Materials and Methods). In both WT and *arm>wg* embryos, the AJs are a fraction lower (∼0.2 µm) at PSB interfaces compared with non-PSB interfaces (Fig. 7G,H). However, this lowering is very small compared with the extent of AJ basal shift observed during dorsal folding in early embryos, where the junctions of dorsal fold cells lower by up to 10 µm, while neighbouring cells shift their AJs by 3 µm (Wang et al., 2012). We conclude that although both involve Baz, epithelial folding at PSBs is likely to occur via a different mechanism than dorsal folding in gastrulating embryos. We then examined apical cell areas at the level of AJs in WT and *arm>wg* embryos; these are, on average, remarkably similar between nonboundary cells and cells adjacent to either endogenous or ectopic boundaries (Fig. 7I,J). Moreover, sampling the sectional areas throughout the 3D volume, we could not find any significant differences (Fig. 7E,F and data not shown), suggesting that, on average, there are no significant changes in cell areas between nonboundary and boundary cells, and that apical cell areas are similar to more basal sectional cell areas. We conclude that the boundary cells at endogenous or ectopic PSBs do not undergo apical constriction. Together, these experiments suggest that both AJ lowering (Wang et al., 2012) and apical constriction (Martin and Goldstein, 2014) do not contribute to PSB fold formation.

Several other mechanisms can be considered. One of them is that the increase in actomyosin tension that we detect at the level of the AJs propagates to the lateral domain, below the AJs. An increase in actomyosin contractility in the lateral interfaces could conceivably shorten those and promote folding. Supporting this notion, an increase in actomyosin contractility at the lateral cortex of ascidian endoderm cells is important for their invagination (Sherrard et al., 2010). We reasoned that if this hypothesis is correct, the lateral surfaces should be straighter at PSBs compared with control lateral interfaces. To test this, we segmented the lateral cell surfaces at endogenous and ectopic boundaries as well as control DV interfaces, in wild-type and *arm>wg* embryos (Fig. S5). We then identified the position of the AJs by the peak of intensities in the corresponding channel, and measured an index of straightness for the *z*-planes above and below the AJs. As expected from previous work, the lateral surfaces are straightest at the level of the AJs and become less straight while moving down basally (Fig. S5A,B) (Monier et al., 2010). But, in addition, lateral surfaces at both endogenous and ectopic boundaries are systematically straighter than control lateral surfaces (Fig. S5A-F″). This suggests that the increased tension identified at the level of AJs does propagate basally in boundary cells. This points towards a role of increased lateral membrane contractility in parasegmental groove formation.

## DISCUSSION

### Actomyosin enrichment at PSBs is required for epithelial folding

In this paper, we demonstrate that the formation of parasegmental grooves requires an actomyosin enrichment at PSBs. Both enrichment and folding occurs at the cell-cell interfaces between *wingless-*expressing cells and *engrailed*-expressing cells, which is the boundary between anterior and posterior compartments in the embryo. In *wingless* mutants, actomyosin enrichments and PSB grooves are both absent (Larsen et al., 2008; Monier et al., 2010; Tetley et al., 2016). When *wingless* is expressed ectopically, either via overexpression using the Gal4 system or in *nkd* mutants, we find an ectopic actomyosin enrichment associated with an ectopic fold in the middle of each parasegment (Fig. 3, Fig. S3). These correlations suggest that parasegmental fold formation requires actomyosin enrichment at the boundaries. Confirming this, inhibition of Myosin II activity using the Rho kinase inhibitor Y-27632 abolishes parasegmental folds, both endogenous and ectopic (Fig. 3K,K′). Thus, inhibition of Myosin II activity is sufficient to disrupt both folding (this study) and boundary straightness (Monier et al., 2010), indicating that these processes are tightly linked. Boundary straightness suggests higher tension along boundary cell-cell contacts, a hallmark of many compartmental and tissue boundaries (Aliee et al., 2012; Amack and Manning, 2012; Calzolari et al., 2014; Fagotto, 2015; Landsberg et al., 2009; Monier et al., 2010; Tetley et al., 2016). Our laser ablations of cell-cell contacts provide direct evidence for this for both endogenous and ectopic PSBs at germ-band extended stages (Fig. 1K, Fig. 3F) and demonstrate that Wingless signalling is required for this increase in tension (Fig. 1L).

Our findings about the regulation of actomyosin enrichments are consistent with what is known about the regulation of parasegmental grooves. Larsen et al. (2008) showed that of the segment polarity genes *wingless*, *engrailed* and *hedgehog*, only *wingless* is required for groove formation, which is what we also find for actomyosin enrichments (Monier et al., 2010; Tetley, 2014; Tetley et al., 2016). Actomyosin enrichments at both ectopic and endogenous PSBs are maintained in embryos expressing *wingless* everywhere, in either a *Dfen^E^* (a small deficiency that removes *engrailed* and its paralogue *invected*) or a *hh^AC^* background (Tetley, 2014) (Fig. 5D).

We also agree with Larsen et al. (2008) that the role of Wingless signalling is permissive rather than instructive. We first detect actomyosin enrichments at PSBs when pair-rule genes are active, during germ-band extension (Tetley et al., 2016). Surprisingly, Wingless is not necessary for actomyosin enrichment at PSBs at these early stages (stages 7-8), but is required later, at germ-band extended stages (9-11) (Monier et al., 2010; Tetley et al., 2016). Moreover, when *wingless* is expressed everywhere, only one ectopic actomyosin enrichment is formed, in the middle of each parasegment. This position corresponds to the boundary between the cells competent to transcribe *engrailed* and those competent to transcribe *wingless* (pink and blue domains in Fig. 3B). These two domains are the result of earlier pair-rule gene activities, in particular the activity of the Sloppy paired transcriptional factors (Cadigan et al., 1994; Clark, 2017; Clark and Akam, 2016; Larsen et al., 2008). We conclude that actomyosin enrichments at PSBs are a consequence of earlier pair-rule transcriptional activities, which are maintained by Wingless signalling at both endogenous and ectopic boundaries.

### Epithelial folding at PSBs is mostly suppressed

Consistent with the above conclusion, we find that some of the planar polarities controlled by pair-rule gene activity during germ-band extension, such as Rok enrichment (Simões et al., 2010) and Baz depletion (Blankenship et al., 2006) are maintained at PSBs at germ-band extended stages, downstream of Wingless signalling. In addition, we find that Flapwing, a component of the Myosin II phosphatase (Vereshchagina et al., 2004), is enriched both at PSB cell-cell interfaces (Fig. 1I) and at junctional and medial actomyosin pools during germ-band extension (Movie 1, Fig. S2). Consistent with the latter localization, a role for Myosin II phosphatase in germ-band extension has recently been demonstrated (Munjal et al., 2015). Later, in germ-band extended embryos, the depletion of Flw increases Myosin II activation at PSB cell-cell interfaces and this correlates with the formation of a deeper fold (Fig. 2). This suggests that although actomyosin enrichment is required for fold formation at PSBs, the folding is mostly suppressed. This fits with the observation that parasegmental folds form almost 2 h after actomyosin enrichments are first detected in early embryos and that even at their most prominent (stage 11), they remain modest indentations of the tissue. The known function of actomyosin enrichments at compartmental boundaries is to provide a mechanical barrier to cell mixing (Aliee et al., 2012; Amack and Manning, 2012; Calzolari et al., 2014; Fagotto, 2015; Landsberg et al., 2009; Monier et al., 2010). In *Drosophila* embryos, cell sorting at AP compartmental boundaries is observed before parasegmental groove formation (Monier et al., 2010; Tetley et al., 2016; Vincent and O'Farrell, 1992). In *Drosophila* wing discs, there is no fold associated with the AP boundary, but in some mutant backgrounds a deep fold can form (Liu et al., 2016; Shen et al., 2008). This suggests that fold formation is not required for compartmental cell segregation and also, that for both embryo and disc AP boundaries, the mechanisms of actomyosin enrichment include pathways suppressing epithelial folding.

In addition to the activity of the phosphatase Flw, fold suppression in the embryo might involve the depletion of Baz at actomyosin enriched cell-cell interfaces. Ectopic boundaries have much deeper folds than endogenous boundaries (Larsen et al., 2008; this study), correlating with a loss of Baz depletion (Fig. 3E). Conversely, when Baz is overexpressed, deep folds form at endogenous PSBs (Fig. 4). Although depletion of Baz requires Wingless signalling, this is counteracted by another signal, Hedgehog (Hh), both at endogenous and ectopic boundaries (Fig. 5E). This suggests that two independent systems, downstream of two distinct signals, have opposite effects on epithelial folding at actomyosin-enriched boundaries. Perhaps Hh signalling is counteracting Wg more effectively later in development, which is why parasegmental folds are most obvious at stage 11 (Martinez-Arias, 1993). Relevant to this, Hh is required from stage 12 onwards for the deep folding at segmental boundaries, located at the posterior edge of the Engrailed-expressing cells (Larsen et al., 2003; Mulinari and Hacker, 2009). This deep folding is inhibited by Wingless signalling at the anterior edge of the *engrailed*-expressing cells, so the antagonistic interaction between Wg and Hh is also relevant to segmental fold formation. At segmental boundaries, Hh signalling is thought to cause apical constriction (Larsen et al., 2003; Mulinari and Hacker, 2009) and this requires Myosin II activation (Mulinari et al., 2008). Interestingly, a role for Hh signalling in Myosin II-dependent apical constriction has also been shown for morphogenetic furrow formation in *Drosophila* eye discs (Corrigall et al., 2007; Escudero et al., 2007). So Wingless signalling might be inhibiting Hh-dependent apical constriction at both parasegmental and segmental boundaries, perhaps by promoting the planar polarization of factors such as Baz.

Our Baz depletion experiments did not abolish the deep folding at ectopic boundaries (Fig. S4C-E), suggesting that Baz is only one of the components in a fold-suppression pathway. An obvious candidate might be Rok as it is known to remove Baz from actomyosin-enriched junctions during germ-band extension (Simões et al., 2010). At PSBs, Rok is enriched and this enrichment requires Wingless signalling like the other polarities (Fig. 1I). At ectopic boundaries, this enrichment increases further (Fig. 3E), so there is no simple relationship between Rok localization and Baz depletion at the PSBs. However, the fact that both factors change at ectopic boundaries might indicate that they are both important in fold regulation and might be co-regulated. Because localization of Rok, however, is not necessarily indicative of its activity, monitoring upstream Rho activity is likely to be more informative (Munjal et al., 2015).

### Epithelial folding at PSBs occurs through a novel mechanism

We propose that two pathways compete at PSBs: Wingless signalling maintains robust actomyosin enrichment at the boundary while preventing folding, whereas Hedgehog signalling promotes folding. The balance between the two would produce shallow folds at endogenous PSBs and deeper folds at ectopic PSBs. To understand the underlying cell biology, we have examined boundary cell behaviours in live and fixed embryos. From analysis of fixed embryos, we cannot detect any significant apical constriction or lowering of AJs in boundary cells (Fig. 7). One caveat of this analysis is that we use embryos at the beginning of fold formation, because of the difficulties of doing image segmentation on already folded embryos. However, our movies of live embryos during folding did not reveal any obvious stereotypical behaviours (Fig. 6). Instead, we observed a range of behaviours (delaminations, cell intercalations, cell elongation along the boundary) suggesting that cells are being displaced and deformed as a consequence of the increased tension at the boundary. We show that boundary tension is not only evident at the level of AJs, but also propagates to the lateral surface (Fig. S5). The simplest hypothesis is that straightening of the PSB lateral surface as a consequence of increased contractility leads to its shortening in the apico-basal axis, producing an indentation in the tissue, explaining the parasegmental grooves. Supporting this, increased lateral contractility in ascidian gastrulation has been shown to cause endoderm cell shortening (Sherrard et al., 2010). Interestingly, a combination of Myosin II-dependent indentation of the epithelium and cell loss through apoptosis underlies folding during leg joint formation in *Drosophila* (Monier et al., 2015). Computational modelling suggests that tissue curvature is important for fold formation in this example. Based on this, it is conceivable that increased lateral contractility, cell displacements at the boundary (Fig. 6) and embryo curvature might all contribute to fold formation at PSBs.

An increase in junctional and/or lateral contractility, however, does not readily explain the presence of deep folds at ectopic PSBs or at endogenous PSBs when Baz is overexpressed. Indeed, we did not find an increase in actomyosin concentration or interfacial tension at the level of the AJs (for example, Fig. 3E,F) and the lateral surfaces at ectopic boundaries are not straighter than those at endogenous boundaries (Fig. S5B). However, we have not measured directly either actomyosin enrichment or tension along lateral surfaces, so we cannot, at present, rule out a difference in these.

Our correlations suggest that levels of Baz along actomyosin-enriched cell-cell contacts could influence epithelial folding. There is growing evidence that Baz affects E-Cadherin dynamics (Bulgakova et al., 2013; Coopman and Djiane, 2016; Truong Quang et al., 2013; Weng and Wieschaus, 2017). E-Cadherin turnover or distribution could be subtly different at endogenous versus ectopic boundaries because of the difference in Baz levels. This could in turn modify the mechanical coupling between E-Cadherin and actomyosin networks (Heer and Martin, 2017; Lecuit and Yap, 2015; Röper, 2015). So, comparable actomyosin enrichments might lead to different deformations, depending on E-Cadherin distribution/turnover. For example, an isotropic distribution of E-Cadherin around the cell is important for effective constriction of apical cell areas (Coravos and Martin, 2016; Lv and Großhans, 2016). More work is needed to understand how the polarization of Baz could affect E-Cadherin dynamics and in turn modify 3D cell shapes. Baz depletion is often associated with actomyosin cable-like enrichments in the absence of folds (Osterfield et al., 2013; Röper, 2013; St Johnston and Sanson, 2011; Tamada and Zallen, 2015), so the suppression of fold formation at actomyosin-enriched boundaries via specific planar asymmetries (here maintained by Wingless signalling and counteracted by Hedgehog signalling) might be relevant beyond PSBs.

## MATERIALS AND METHODS

### Fly strains

The mutant alleles and constructs used in this study are listed in the supplementary Materials and Methods. Genotypes are provided in Table S1. Gene information is from FlyBase (Gramates et al., 2017).

### Immunostaining

Standard protocols were used to fix embryos and are described in full, along with a list of primary antibodies, in the supplementary Materials and Methods.

### PSB enrichment imaging and analysis

Fixed embryos were imaged by standard confocal optical sectioning and enrichment was analysed in stage 10 embryos, except for *arm>wg*, for which late stage 9 embryos were used to avoid too much folding at the ectopic boundaries. Quantification of protein enrichment was performed in maximum intensity projections of *z* optical slices containing the AJs. Three-pixel-wide traces along PSBs or control DV-oriented cell-cell contacts were drawn using ImageJ plug-ins. Average fluorescence intensities from the traces were background subtracted first, then PSB enrichment or depletion was calculated by dividing the PSB fluorescence intensity for a given marker by the fluorescence intensity for a nearby control DV-oriented trace. The log_10_ of these ratios were plotted to restore symmetry between enrichments and depletions on the plots and facilitate statistical comparisons.

### 3D image segmentation, quantification of cell areas, AJ position and index of straightness

Stage 10 wild-type and *arm>wg* embryos were fixed and immunostained for En, E-Cadherin and phalloidin to label the PSBs, AJs and cell contours, respectively. Note that *arm>wg* embryos were a little younger than wild type to minimize folding at ectopic boundaries; earlier staging is evident from the midline cells not yet being invaginated in *arm>wg* embryos (Fig. S5E,E″), in contrast to wild-type embryos (Fig. S5C,C″) (Martinez-Arias, 1993).

Embryos were imaged by standard confocal imaging, optically sectioning from the top to the bottom of the cell. The actin phalloidin signal was used to segment the 3D shapes of the cells. Segmented cells either abutting the endogenous or ectopic boundaries, or away from the boundaries as controls, were selected for quantitative analysis. Cell areas were quantified for each optical slice, including an apical slice corresponding to the AJs presented in Fig. 7I,J.

For measuring AJ positions and indices of straightness, line traces were generated at the level of AJs as for quantifying PSB enrichment/depletion. These traces were propagated to find the cells' apico-basal surfaces above and below AJs (dividing cells were discarded). An estimate of the cell top was calculated and used to measure the distance between top of the cell and AJ position, for each cell. An index of straightness was calculated for each line trace propagated at each *z* position above and below AJs.

### Scanning electron microscopy

Embryos were fixed, dehydrated and gold coated following standard protocols, then imaged with the scanning electron microscopy (SEM) from the Cambridge Advanced Imaging Centre (CAIC).

### Embryo drug injections

Rok inhibitor Y-27632 at 1 mM was injected into the yolk of early stage 9 *arm>wg* embryos. We established previously that, presumably because of the whole embryo dilution, this concentration is not sufficient to block cell division, but is sufficient to disrupt boundary formation because PSBs lose their characteristic straightness (Monier et al., 2010). Embryos were aged until stage 10, then fixed and manually devitellinized. A secondary fixation was performed to process the embryos for SEM as above.

### Live imaging

Embryos were dechorionated in bleach and imaged in halocarbon oil, using either a spinning disc confocal (Fig. 6) or for performing ablations, a two-photon confocal microscope, see below.

### Laser ablation

A near-infrared laser delivering femtosecond pulses coupled to a two-photon confocal microscope (in CAIC), was used to perform ablations in embryos carrying *sqh-GFP* in different genotypes (Table S1). Line ablations ∼2 μm long were performed in the middle of a cell-cell contact at the level of AJs. Kymographs imaging *sqh-GFP* signal before and after ablation were used to quantify the recoil velocity of cut ends over time.

Further details for each of the above sections are provided in the supplementary Materials and Methods.

## Supplementary Material

Supplementary information

Supplementary information
