# Supplementary material for: Suppression of epithelial folding at actomyosin-enriched compartment boundaries downstream of Wingless signalling in Drosophila
Source: Development. 2018 Apr 24;145(8):dev155325. doi: 10.1242/dev.155325 (PMC5964650; doi:10.1242/dev.155325)
Supplement: Supplementary information [file develop-145-155325-s1.pdf]

Supplementary Materials and Methods

<u>*Drosophila* strains</u>

The table below lists the alleles used and Table S1 the genotypes for each figure.

Name	Genotype	Origin
yw	y^1w^{67c23}	Bloomington #6599
wg^{CX4}	wg^{CX4}	Baker, 1987
enlacZ	en-lacZ	Busturia and Morata, 1988
rokGFP	Ubi-Rok::GFP	Gift from Vincent Mirouse
flwYFP	$flwYFP^{CPTI-002264}$	Lowe et al,, 2014; Lye et al.,2014
bazGFP	$bazGFP^{CC01941}$	Buszczak et al., 2007
prdGal4	prd-Gal4	Bloomington #1947 (Brand & Perrimon 1993)
UASdeGradFP	UAS-deGradFP	Caussinus et al., 2011
armGal4	arm-Gal4	Sanson et al., 1996
UASwg	UAS-wg	Lawrence et al., 1995
MTDGal4	otu-GAL4::VP16, w^*; GAL4-nos.NGT; GAL4::VP16-nos.UTR	Bloomington #31777 (Petrella et al., 2007)
UASbazGFP	UASp-baz::GFP	Benton & St Johnston 2003
hh^{AC}	hh^{AC}	Lee et al., 1992
sqhGFP40	sqhGFP40 (III)	Royou et al., 2002
eveGFP	eve::EGFP (III)	Venken et al., 2009
Gap43Cherry	$GAP43^{mem}$::mCherry	Rauzi et al., 2010
baz^{XR11}	baz^{XR11}	Kuchinke et al., 1998
nkd^2	nkd^2	Tearle & Nüsslein-Volhard 1987
rhoGal4	rho-Gal4	Ip et. al. 1992
CyO TwistG4 UAS EGFP (CTG)	CyO, P{w[+mC]=GAL4-twi.G}2.2, P{UAS-2xEGFP}AH2.2	Bloomington #6662 (Halfon et al 2002)
CyOwglacZ	$CyO-P\{en1\}wg^{en11}$	Heemskerk & DiNardo 1994
TM6C Twi LacZ (TTLZ)	TM6C, P{w[+mW.hs]=twi-betaGal-1.4t}LS1, Sb[1] Tb[1]	Bloomington #7251 (Seugnet et al 1997)

<u>Immunostainings</u>

Embryonic staging was as in (Hartenstein and Campos-Ortega, 1985). Embryos were collected in a basket from one-hour collections on plates containing apple or grape juice hardened with agar. They were dechorionated by immersion in commercial bleach diluted 1:2 in pure water, for 2 minutes, rinsed, blotted dry and then transferred into heptane. For most experiments, embryos were fixed for 5 minutes in the interface of a 1:1 solution of Heptane:Formaldehyde 37% followed by manual devitelinization in PBS with 0.1% Triton X-100 in (PTX). For staining against phospho-Moesin, 10% trichloroacetic acid in dH_2O was used instead of the formaldehyde, and the embryos fixed on ice for 1 hour. Embryos were then blocked with PTX containing 1% bovine serum albumin (PTB) for 30 minutes, and incubated overnight at 4ºC with primary antibodies. They were washed three times for 15 minutes in PTX, then incubated for one hour with secondary antibodies in PTB. They were washed a further three times in PTX, and stored at -20ºC in Vectashield (Vector laboratories). When biotin-conjugated secondary antibodies were used an extra step was used. After the second antibody washes the embryos were incubated with streptavidin- conjugated Alexa-405 for 30 minutes before three further washes in PTX, and stored at -20ºC in Vectashield.

Antibodies

The following monoclonal primary antibodies were obtained from the Developmental Studies Hybridoma Bank (NICHD and NIH; University of Iowa, Department of Biology, Iowa City): mouse anti-En (4D9; 1:100, Goodman 1989), rat anti-DE-cadherin (DCAD2; 1:50, Takeichi 1994), mouse anti-Dlg (4F3; 1:500, Parnas et al., 2001), rat anti-alpha-catenin (DCAT-1; 1:100, Takeichi 1993), mouse anti-Wingless (4D4; 1:50, Brook & Cohen 1996). Other primary antibodies were: rabbit anti-Baz (1:500; a gift from A. Wodarz), chicken anti-ß-Gal (Abcam ab9361; 1:500), mouse anti-ß-Gal (Promega Z3781, 1:5000), rabbit anti-β-gal (MP Biomedicals #559761; 1:2500), rabbit anti-Engrailed (Santa Cruz Biotechnology d-300; 1:200), goat anti-GFP-FITC (Abcam ab6556, 1:200), guinea pig anti-Sqh-1P (1:100, a gift from R.E. Ward IV), mouse anti-phospho-Tyrosine (Cell signaling #9411; 1:100), rabbit anti-phospho-Ezrin/Radixin/Moesin (Cell Signalling #3141, 1:200).

Secondary antibodies conjugated to fluorescent dyes were obtained from Jackson ImmunoResearch Laboratories, Invitrogen and Life Technologies. Streptavidin with Alexa Fluor 405 conjugate was from ThermoFisher Scientific. Cell nuclei were stained using Vectashield with DAPI (Vector Laboratories). F-actin was stained using CF594-conjugated phalloidin (Biotium #00045; 1:1,000).

Confocal imaging

Embryos were mounted individually under a coverslip supported by a tape bridge on either side. This flattened the embryos sufficiently so that all cells were roughly in the same z-plane. Embryos were imaged on a Nikon Eclipse TE2000 microscope incorporating a C1 Plus confocal system (Nikon) and images captured using Nikon EZ-C1 software; or, a Leica TCS SP8 confocal microscope and images captured using LAS X software (Leica). Optical z-stacks were acquired with a depth of 0.25 µm between successive optical z-slices. All embryos were imaged using a violet corrected 60x oil objective lens (NA of 1.4). The gain and offset were optimized for each embryo.

Quantification of enrichment at PSBs

Two stages were used for quantification: stage 10 embryos in all genotypes except for *arm>wg*, where late stage 9 embryos were analyzed to avoid too much folding at ectopic boundaries. Quantifications were done in maximum intensity projections, which were made from the minimum number of z-slices needed to contain all the adherens junction signal. The adherens junctions were labelled by staining for either E-Cadherin or phospho-Tyrosin. Cortical signal of different proteins was quantified on line traces that went over cell interfaces. The position of the PSB was identified by co-staining with anti-En or anti-Wg, except for wg^{CX4} embryos in which these markers are gradually lost; in this case, an *enlacZ* transgene was used and staining with anti-ßGal showed the PSB location (ßGal protein has longer perdurance than En protein in embryos). The lines were manually traced by using the FIJI plugin Simple Neurite Tracer (Longair et al., 2011) or the ImageJ plugin NeuronJ (Meijering et al., 2004) based on membrane marker stainings and avoiding dividing cells. Average fluorescence intensity was quantified for 3-pixel wide line traces using ImageJ or FIJI (Schneider et al, 2012; Schindelin et al, 2012). We used the image-wide modal pixel intensity as an approximation of the average background fluorescence. The modal pixel intensity was then subtracted from all pixels to remove background fluorescence from the signal. PSB and Ectopic Boundary interface fluorescence intensity was then normalised to En interface fluorescence intensity for each PSB quantification (Example in Figure 1 D", G"), with the exception of Baz, for which it was normalized to DV tracks outside the Engrailed domain (Example in Figure 1E", H"). This is because contrarily to the other proteins we looked at, Baz shows a very weak remaining planar polarity at stage 10, in particular in the Engrailed domain. Statistics were performed in Prism (GraphPad). Pilot experiments were used to establish that $n \approx 20$ PSBs was appropriate for the detection of enrichments or depletions. Data from all quantifications are reported as mean ± 95% confidence intervals. Results were considered significant when $p < 0.05$ (* when $p < 0.05$, ** when $p < 0.01$, *** when $p < 0.001$, **** when $p < 0.0001$).

Development • Supplementary information

3D image segmentation, quantification of cell areas, AJ position and index of straightness

Wildtype and *arm>wg* tage 10 embryos were stained with Engrailed and E-Cadherin antibodies as well as CF594-Phalloidin to mark PSBs, adherens junctions and actin respectively. Then, embryos were mounted under a coverslip suspended by a two-layer thick tape bridge on either side. The samples were imaged on a Leica TCS SP8 confocal microscope (CAIC, University of Cambridge). Optical z-stacks were acquired with a depth of 0.33 µm between successive optical z-slices, which is the optimal z interval thickness of the 63X objective used. The gain and offset were optimized for each embryo. Fluorescence images were denoised (Boulanger et al., 2010) and segmented using Real-time Accurate Cell-shape Extractor (Stegmaier et al, 2016). Cell top was detected by the apical medial actin enrichment while cortical actin decorated cell contour. Segmented images were used in ImageJ to manually select cells of different populations (Control, PSB and ectopic PSBs: ECT) in wildtype and *arm>wg* embryos. Selected cells were saved as region of interests and used to quantify cell area per stack and 3D render. Custom written MATLAB scripts computed cell areas for the chosen cells in each plane of the stack.

For the adherens junctions apico-basal position, analysis contours were generated as described above for the quantification of protein enrichments at PSBs and saved as 2D binary masks. The cell walls corresponding to the regions of interest were determined by propagating these contours as open snakes on the cortical Phalloidin channel intensities (Shemesh and Ben-Shahar, 2001). These cell walls were then used to quantify the distance between the adherens junctions (E-cadherin) and the top of the cell, detected by medial actin (Phalloidin). The positions of the adherens junctions were given by the maxima of E-cadherin channel values in z direction along the wall. An estimate of the top of the cell was obtained by segmenting the Phalloidin channel stack in 2D (xz direction) via robust statistics based thresholding of the wavelet coefficients of the image. 2D projections of intensities in the E-cadherin and Phalloidin channel (across the width of the bounding box for each input contour) were saved as a mean of quality control by visual inspection. The distance between adherens junctions and the closest point of the cell top was computed taking into account voxel anisotropy. Finally, as a post-processing step of removing outliers, the highest 10% of distances were discarded for each region.

The index of straightness (IS, (Monier et al., 2010) was computed for each propagated contour in each plane of the 3D stack over a depth of 5 microns (starting from 0.6 microns above the adherens junctions). It is calculated as representing the percentage of curve length exceeding the length of the straight line joining the curve's endpoints:
IS = (length of curve / distance between the two endpoints of the curve - 1) * 100

Scanning Electron microscopy

Embryos were fixed for 5 minutes in Heptane:Formaldehyde 37% (1:1) and devitellinised with Heptane:Methanol (1:1). Then, they were re-fixed immediately in 2% Glutaraldehyde, 2% Formaldehyde, 0.05M Sodium Cacodylate pH 7.4 and 2mmol/L Calcium Chloride overnight. Once rinsed twice in deionised water, embryos were treated with 1% osmium ferricyanide for 3 days. After that they were rinsed four times in deionised water, dehydrated to 100% ethanol and dried by either critical point drying, or drying from hexamethyldisilazane (HMDS). Where HMDS was used, embryos were transferred into 1:1 HMDS:ethanol for 10 minute, then HMDS for 10 minutes twice, and left to dry. Dry embryos were mounted on carbon tabs on 12.5 mm Cambridge stubs and sputter coated with 50nm of gold. Images were taken in a FEI XL30 FEG scanning electron microscope operated at 5 kV.

Y-27632 Rho kinase inhibitor injections

Early stage 9 *arm>wg* embryos were injected through the posterior into the yolk at room temperature with 1 mM Y27632 in dH_2O, and dH_2O in control experiments. Embryos were aged for 45 minutes at 25°C, then fixed in 8% formaldehyde over heptane for 20

Development • Supplementary information

minutes. They were rinsed with PBS, manually devitellinised by nicking with a needle, and then fixed for SEM as above.

Live Imaging

Dechorionated embryos were transferred into halocarbon oil (Voltalef PCTFE, Arkema), mounted ventral side up on stretched oxygen-permeable membrane, and covered with a coverslip supported by a bridge of a single coverslip on either side. Timelapse imaging was carried out on a Nikon Eclipse E1000 equipped with a spinning disk unit (Yokogawa CSU10), laser module with 491nm and 561nm excitation (Spectral Applied Research LMM2), and a C9100-13 EM-CCD camera (Hamamatsu). Z-stacks were acquired with an interval of 0.7µm or 1µm. Images were captured using Volocity software (PerkinElmer). Where multiple images were stitched together (Fig S4C), the FIJI plugin Grid/collection Stitching was used (Preibisch et al., 2009).

Laser ablations and analysis of recoil velocities

Laser ablation experiments were carried out on a TriM Scope II Upright 2-photon Scanning Fluorescence Microscope controlled by Imspector Pro software (LaVision Biotec) using a tuneable near-infrared (NIR) laser source delivering 120 femtosecond pulses with a repetition rate of 80 MHz (Insight DeepSee, Spectra-Physics). The laser was tuned to 927nm, with power ranging between 1.40-1.70 W. The maximum laser power allowed to reach the sample was set to 220 mW and an Electro-Optical Modulator (EOM) was used to allow microsecond switching between imaging and treatment laser powers. The laser light was focused by a 25x, 1.05 Numerical Aperture (NA) water immersion objective lens with a 2mm working distance (XLPLN25XWMP2, Olympus). Images were collected every 0.731 ms for 5 frames before the ablation and 60 frames after the ablation.

Ablations were performed during image acquisition (with a dwell time of 9.27 µsec per pixel), with the laser power switching between treatment and imaging powers as the laser was raster scanned across the sample. Targeted line ablations of about 2 µm length were performed at the centre of junctions on the PS boundary or on control, non boundary dorso-ventral (DV) oriented or antero-posterior (AP) oriented junctions, using a treatment power of 220 mW. 20-25 ablations per condition per genotype were carried out, 2-4 ablations per embryo.

To analyse recoil velocities, a kymograph spanning the ablated region was extracted using the dynamic reslice function in Fiji, and the distance between the two ends of the cut was measured up to 30 seconds after ablation. Linear regression was performed on the first 5 timepoints after ablation and the slope of the regressed line was used to measure the recoil velocity of the cut ends.

References:

Baker, N.E. (1987). Molecular cloning of sequences from wingless a segment polarity gene in Drosophila the spatial distribution of a transcript in embryos. EMBO J. 6(6): 1765--1774.

Benton, R. & St. Johnston, D. (2003). A conserved oligomerization domain in Drosophila Bazooka/PAR-3 is important for apical localization and epithelial polarity. Curr. Biol. 13(15): 1330--1334.

Boulanger, J., Kervrann, C., Bouthemy, P., Elbau, P., Sibarita, J. B. and Salamero, J. (2010). Patch-based nonlocal functional for denoising fluorescence microscopy image sequences. *IEEE transactions on medical imaging* 29, 442-454.

Brand, A.H., Perrimon, N. (1993). Targeted gene expression as a means of altering cell fates

Development • Supplementary information

and generating dominant phenotypes. Development 118(2): 401--415.

Brook W.J. & Cohen SM. (1996). Antagonistic interactions between wingless and decapentaplegic responsible for dorsal-ventral pattern in the Drosophila Leg. Science 273(5280): 1373-7.

Busturia, A., Morata, G. (1988). Ectopic expression of homeotic genes caused by the elimination of the Polycomb gene in Drosophila imaginal epidermis. Development 104: 713--720.

Buszczak, M., et al. (2007). The Carnegie protein trap library: A versatile tool for Drosophila developmental studies. Genetics 175(3): 1505--1531.

Caussinus, E., et al. (2011). Fluorescent fusion protein knockout mediated by anti-GFP nanobody. Nat. Struct. Mol. Biol. 19(1): 117--121.

Goodman CS. (1989). Expression of engrailed proteins in arthropods, annelids, and chordates. Cell 58(5): 955-68.

Halfon, M.S., et al. (2002). New fluorescent protein reporters for use with the Drosophila Gal4 expression system and for vital detection of balancer chromosomes. genesis 34(1-2): 135--138.

Hartenstein, V. and Campos-Ortega, J. A. (1985). Fate-mapping in wild-type Drosophila melanogaster. 1. The spatio-temporal pattern of embryonic cell divisions. *Roux's Arch. Dev. Biol.* 194, 181-195.

Heemskerk, J. & DiNardo, S. (1994). Drosophila hedgehog acts as a morphogen in cellular patterning. Cell 76(3): 449--460.

Ip, T.Y., et al. (1992). The dorsal gradient morphogen regulates stripes of rhomboid expression in the presumptive neuroectoderm of the Drosophila embryo. Genes Dev. 6, 1728-1739.

Kuchinke, U., et al. (1998). Control of spindle orientation in Drosophila by the Par-3-related PDZ-domain protein Bazooka. Curr. Biol. 8(25): 1357--1365.

Lawrence, P.A., et al. (1995). Segmental patterning of heart precursors in Drosophila. Development 121(12): 4303--4308.

Lee, J.J., et al. (1992). Secretion and localized transcription suggest a role in positional signaling for products of the segmentation gene hedgehog. Cell 71(): 33--50.

Longair, M. H., Baker, D. A. and Armstrong, J. D. (2011). Simple Neurite Tracer: open source software for reconstruction, visualization and analysis of neuronal processes. Bioinformatics 27, 2453–2454.

Lowe, N., et al. (2014). Analysis of the expression patterns, subcellular localisations and interaction partners of Drosophila proteins using a pigP protein trap library. Development 141(20): 3994--4005.

Lye, C.M., et al. (2014). Subcellular localisations of the CPTI collection of YFP-tagged proteins in Drosophila embryos. Development 141(20): 4006--4017.

Development • Supplementary information

Meijering E., et al. (2004). Design and Validation of a Tool for Neurite Tracing and Analysis in Fluorescence Microscopy. Cytometry A. 58(2):167-76.

Monier, B., Pelissier-Monier, A., Brand, A. H. and Sanson, B. (2010). An actomyosin-based barrier inhibits cell mixing at compartmental boundaries in Drosophila embryos. *Nat Cell Biol* 12, 60-65.

Parnas D, Haghighi AP, Fetter RD, Kim SW, Goodman CS. (2001). Regulation of postsynaptic structure and protein localization by the Rho-type guanine nucleotide exchange factor dPix. Neuron. 32(3):415-24.

Petrella, L.N., et al. (2007). The Ovhts polyprotein is cleaved to produce fusome and ring canal proteins required for Drosophila oogenesis. Development 134(4): 703--712.

Preibisch S., et al. (2009). Globally optimal stitching of tiled 3D microscopic image acquisitions. Bioinformatics 25(11): 1463–1465.

Rauzi, M., et al. (2010). Planar polarized actomyosin contractile flows control epithelial junction remodelling. Nature 468(7327): 1110--1114.

Royou, A., et al. (2002). Cortical recruitment of nonmuscle myosin II in early syncytial Drosophila embryos: its role in nuclear axial expansion and its regulation by Cdc2 activity. J. Cell Biol. 158(1): 127--137.

Sanson, B., et al. (1996). Uncoupling cadherin-based adhesion from wingless signalling in Drosophila. Nature 383(6601): 627--630.

Schindelin, J., et al. (2012). Fiji: an open-source platform for biological-image analysis. Nature methods 9(7): 676-682,

Schneider, C.A., et al. (2012). NIH Image to ImageJ: 25 years of image analysis. Nature methods 9(7): 671-675

Seugnet, L., et al. (1997). Transcriptional regulation of Notch and Delta: requirement for neuroblast segregation in Drosophila. *Development* 124(10): 2015--2025.

Shemesh M. & Ben-Shahar O. (2011) Free Boundary Conditions Active Contours with Applications for Vision. In: Bebis G. et al. (eds) Advances in Visual Computing. ISVC 2011. Lecture Notes in Computer Science, vol 6938. Springer, Berlin, Heidelberg

Stegmaier J, et al. (2016). Real-Time Three-Dimensional Cell Segmentation in Large-Scale Microscopy Data of Developing Embryos. Dev Cell. 36(2):225-40.

Takeichi M. (1993). Identification of a Drosophila homologue of alpha-catenin and its association with the armadillo protein. The Journal of cell biology 121(5): 1133-40.

Takeichi M. (1994). A Drosophila homolog of cadherin associated with armadillo and essential for embryonic cell-cell adhesion. Developmental biology 165(2): 716-26.

Tearle, R.G. & Nusslein-Volhard, C. (1987). Tubingen mutants and stock list. D. I. S. 66(): 209--269.

Venken, K.J.T., et al. (2009). Versatile P[acman] BAC libraries for transgenesis studies in Drosophila melanogaster. Nat. Methods 6(6): 431--434.

Figure	Panel	Parental genotype(s)	Embryo genotype
1	A, D-D”, E-E”, I	yw	yw
1	B, H-H”	wg^{CX4}/CTG	wg^{CX4}/wg^{CX4}
1	G-G”, I	wg^{CX4}, en-lacZ/CTG x wg^{CX4}/CTG	wg^{CX4}, en-lacZ/wg^{CX4}
1	I	Ubi-Rok::GFP/TM3Sb	Ubi-Rok::GFP/TM3Sb
1	I	flwYFP	flwYFP
1	I	wg^{CX4}/CTG; Ubi-Rok::GFP/TTLZ x wg^{CX4}, en-lacZ/CTG	wg^{CX4}, en-lacZ/wg^{CX4}; Ubi-Rok::GFP/+
1	I	☿flwYFP; wg^{CX4}/CTG x ♂ wg^{CX4}, en-lacZ/CTG	flwYFP/+ ; wg^{CX4}/wg^{CX4}
1	J, J’, K	w;; sqhGFP40	w;; sqhGFP40
1	L	wg^{CX4}/CTG; sqhGFP40	wg^{CX4}/ wg^{CX4}; sqhGFP40
2	A, B-B”, D, D’, E, F, H-H”	flwYFP;; prdGal4/TTLZ x flwYFP;; UAS-deGradFP/UAS-deGradFP	flwYFP;; prdGal4/UAS-deGradFP
2	C, E	flwYFP;; prdGal4/TTLZ x flwYFP;; UAS-deGradFP/UAS-deGradFP	flwYFP;; TTLZ/UAS-deGradFP
3	D-D”, E, H-H”, J-J’, K-K’	☿armGal4 x ♂ UAS-wg	armGal4/+; UAS-wg/+
3	I	yw	yw
3	E	☿armGal4/+; Ubi-Rok::GFP/+ x ♂ UAS-wg	armGal4/+; Ubi-Rok::GFP/UAS-wg
3	E	flwYFP; armGal4 x flwYFP;; UAS-wg	flwYFP; armGal4/+; UAS-wg/+
3	F	☿armGal4; sqhGFP40 x ♂ UAS-wg	armGal4/+; UAS-wg/sqhGFP40
4	B-B’, D-D”, E, F	☿MTDGal4/UAS-bazGFP x ♂ UAS-bazGFP	UAS-bazGFP/(UAS-bazGFP or MTDGal4)
4	C	☿MTDGal4/UAS-bazGFP; sqhGFP40 x ♂ UAS-bazGFP; sqhGFP40	UAS-bazGFP/(UAS-bazGFP or MTDGal4); sqhGFP40/(sqhGFP40 or MTDGal4)
4	E, F	yw	yw
5	B, D, E	☿armGal4 x ♂ UAS-wg	armGal4/+; UAS-wg/+
5	C, D, E	☿armGal4; hh^{AC}/ TTLZ x ♂ UAS-wg, hh^{AC}/ TTLZ	armGal4/UAS-wg ; hh^{AC}/ hh^{AC}
6	A-A’”	sqh^{AX3}; sqhGFP42; Gap43-mCherry/TM6B	sqh^{AX3}; sqhGFP42; Gap43-mCherry/TM6B
6	B-B’”	☿armGal4/CTG; Gap43-mCherry, eve-GFP/TM6B x ♂UAS-wg	armGal4/+; Gap43-mCherry, eve-GFP/UAS-wg
7	A-A’, C, E, G, I	yw	yw
7	B-B’, D, F, H, J	☿armGal4 x ♂ UAS-wg	armGal4/+; UAS-wg/+
S1	A-A”	yw	yw

S1	B-B”	*Ubi-Rok::GFP/TM3Sb*	*Ubi-Rok::GFP/TM3Sb*
S1	C-C”	*flwYFP*	*flwYFP*
S1	D-D’	*w;; sqhGFP40*	*w;; sqhGFP40*
S1	E-E’	wg^{CX4}*/CTG; sqhGFP40*	wg^{CX4}*/* wg^{CX4}*; sqhGFP40*
S2	A-A”, B-B”, C-C”	*flwYFP*	*flwYFP*
S2	D	*flwYFP;; prdGal4/TTLZ*<br>*x flwYFP;; UAS-deGradFP/UAS-deGradFP*	*flwYFP;; prdGal4/UAS-deGradFP*
S3	A-A”	*☿armGal4 x ♂ UAS-wg*	*armGal4/+; UAS-wg/+*
S3	B-B’	*☿armGal4; sqhGFP40 x ♂ UAS-wg*	*armGal4/+; UAS-wg/sqhGFP40*
S3	D-D’, E-E”	*☿rhoGal4 x ♂UAS-wg*	*rhoGal4/+; UAS-wg/+*
S3	G-G’, H-H”	nkd^{2}*/TTLZ*	nkd^{2}*/*nkd^{2}
S4	A	*yw*	*yw*
S4	A	*☿MTDGal4/UAS-bazGFP x ♂ UAS-bazGFP*	*UAS-bazGFP/(UAS-bazGFP or MTDGal4)*
S4	B-B’	*☿MTDGal4/UAS-bazGFP; sqhGFP40*<br>*x ♂ UAS-bazGFP; sqhGFP40*	*UAS-bazGFP/(UAS-bazGFP or MTDGal4);*<br>*sqhGFP40/(sqhGFP40 or MTDGal4)*
S4	C	*☿bazGFP;; prdGal4/TM6B x ♂ bazGFP; UAS-deGradFP*	*bazGFP/bazGFP; UAS-deGradFP/+; prdGal4/+*
S4	D	*☿bazGFP/*baz^{XR11}*; armGal4 x ♂ bazGFP; UAS-deGradFP/CyOwglacZ; UAS-wg/TM6B*	*bazGFP/*baz^{XR11}*; armGal4/UAS-deGradFP; UAS-wg/+*
S4	E	*☿bazGFP; armGal4 x ♂ bazGFP; UAS-deGradFP/CyOwglacZ; UAS-wg/TM6B*	*bazGFP/bazGFP; armGal4/UAS-deGradFP; UAS-wg/+*
S5	A, C-C”, D-D”	*yw*	*yw*
S5	B, E-E”, F-F”	*☿armGal4 x ♂ UAS-wg*	*armGal4/+; UAS-wg/+*

Table S1: List of genotypes used in figures

Development • Supplementary information

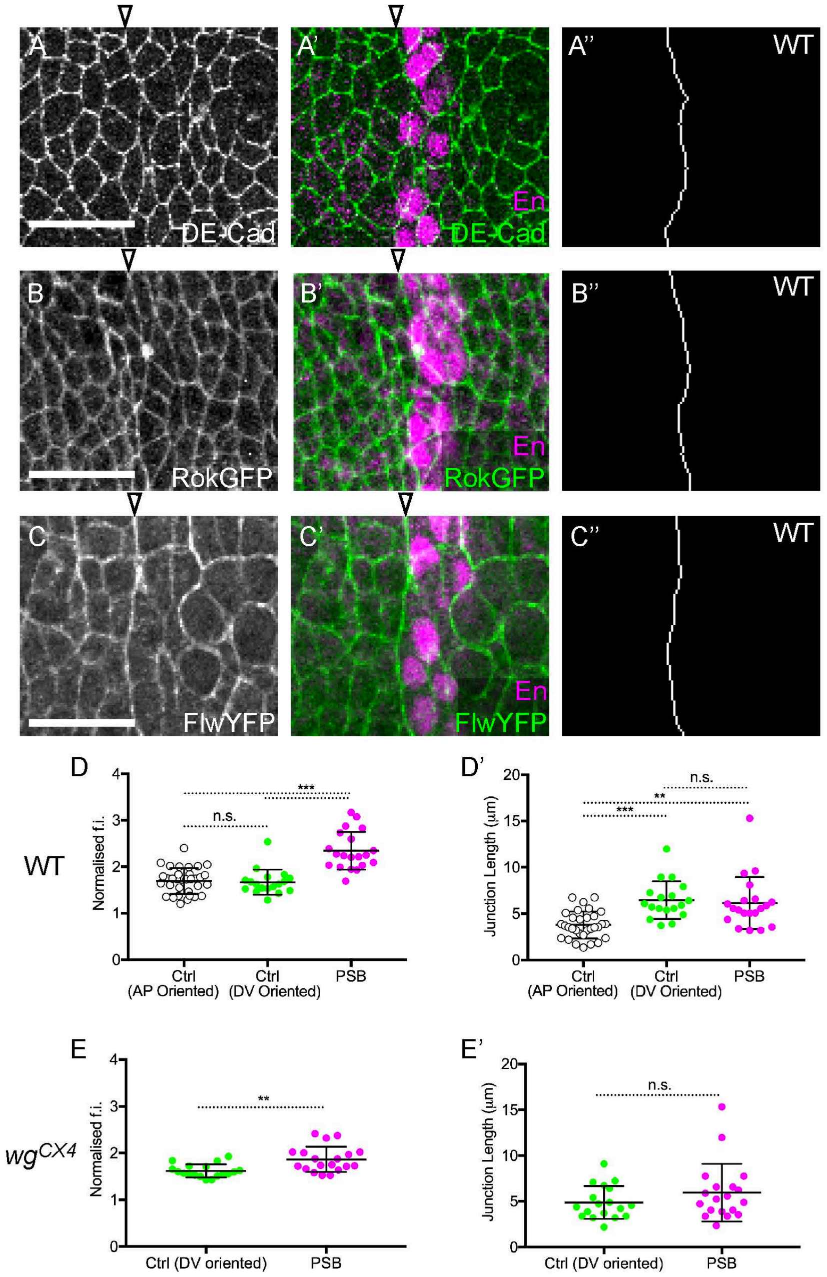

Figure S1: Planar polarities and actomyosin contractility at the PSBs. (A-C”) Example immunostainings used for quantification in Fig. 1I. (A,B,C) Immunostaining against each marker; (A’,B’,C’) merged with Engrailed marker staining to locate the PSBs (open arrowheads). (A”,B”,C”) Tracings along PSB cell-cell junctions. (D-E’) Controls for laser ablations shown in Fig. 1J-L: Myosin II intensity and junction length. (D,E) Quantification of Myosin-II fluorescence intensity (f.i.) using the Sqh-GFP signal at ablated PSB and control junctions in (D) wildtype and (E) wg^{CX4} embryos. Comparisons in (D) from a Kruskal-Wallis Test: AP controls vs. DV controls: p>0.999, n.s.; AP controls vs. PSBs: p<0.0001****; DV controls vs. PSBs: p<0.0001****. Comparison in (E) from a t-test: p=0.0015**. (D’,E’) Length of the ablated PSB and control cell-cell junctions. DV-oriented junctions (PSB and control) are longer than AP-oriented junctions as cells tend to be DV-elongated at this stage. This length difference does not appear to affect recoil speed (see Fig. 1K). Error bars show mean±s.d. Comparisons in (D’) from a Kruskal-Wallis Test: AP controls vs. DV controls: p<0.0001****; AP controls vs. PSBs: p=0.0016**; DV controls vs. PSBs: p>0.999, n.s. Comparison in (E’) from a Mann-Whitney test: p=0.316, n.s.

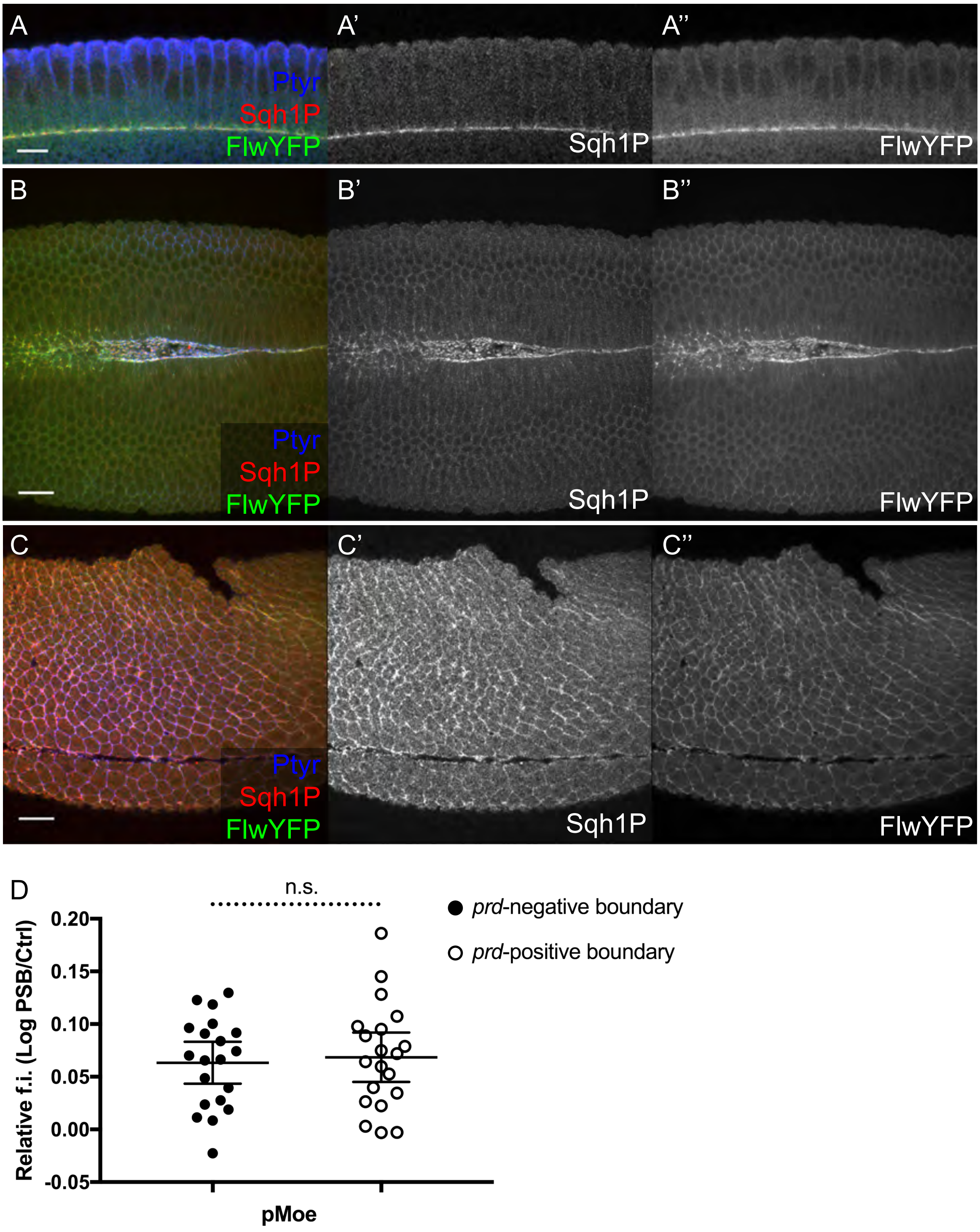

Figure S2: Localisation of Flapwing in early embryos and pMoesin quantification at Flapwing-depleted parasegment boundaries. (A-C") Immunostainings against Flapwing-YFP (Flw-YFP) and monophosphorylated Sqh (Sqh1P) in early embryos. (A-A") Sagittal view of an embryo during cellularisation. (B-B") View of the invaginating mesoderm during gastrulation. (C-C") View of the extending germband of a stage 7 embryo. (A",B",C") anti-GFP staining to label Flw-YFP; (A',B',C') Sqh1P staining; (A,B,C) merged channels with the adherens junction marker phosphoTyrosine (pTyr). Scale bars: 20µm. (D) Quantification of fluorescence intensity (f.i.) of phospho-Moesin (pMoe) along PSBs in deGradFP-expressing and -non-expressing domains (prd-Gal4 positive or negative), relative to control cell interfaces, as $\log_{10}$. n=20 PSBs for both types. Error bars show mean±95% c.i. Comparison from a Mann-Whitney test: p=0.925, n.s.

Development • Supplementary information

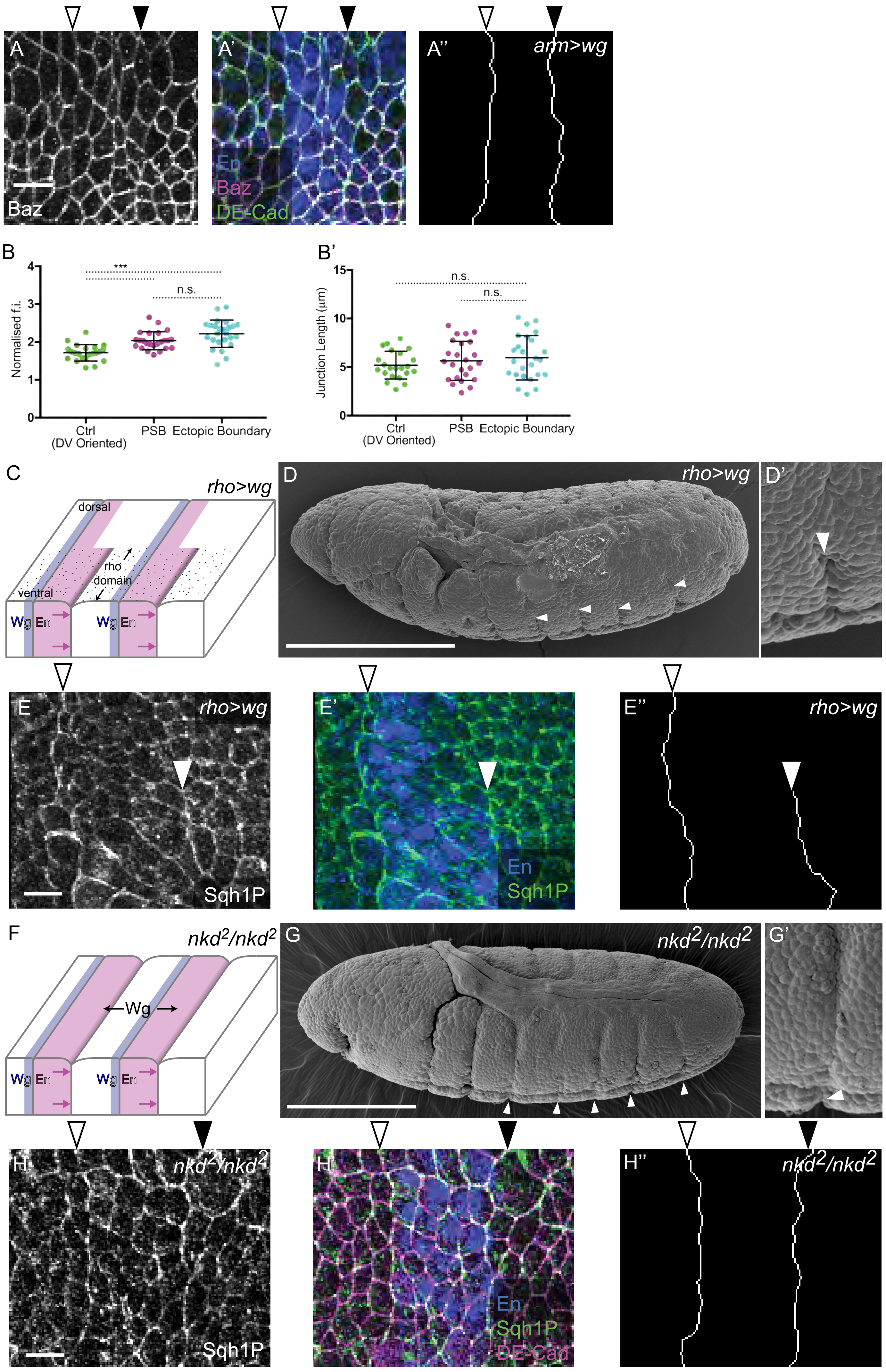

A
Baz
A'
En
Baz
DE-Cad
A''
arm>wg
B
Normalised f.i.
***
n.s.
Ctrl (DV Oriented)
PSB
Ectopic Boundary
B'
Junction Length (μm)
n.s.
n.s.
Ctrl (DV Oriented)
PSB
Ectopic Boundary
C
rho>wg
dorsal
rho domain
ventral
Wg En
Wg En
D
rho>wg
D'
E
rho>wg
Sqh1P
E'
En
Sqh1P
E''
rho>wg
F
nkd2/nkd2
Wg
Wg En
Wg En
G
nkd2/nkd2
G'
H
nkd2/nkd2
Sqh1P
H'
En
Sqh1P
DE-Cad
H''
nkd2/nkd2

Figure S3: Ectopic PSBs in embryos ectopically expressing Wingless. (A-A') Immunostaining of *arm>wg* stage 10 embryo against (A) Bazooka (Baz), (A') merged with Engrailed (En) and E-Cadherin (DE-Cad). (A") Tracings of cell-cell contacts at endogenous and ectopic PSBs. Scale bar: 10µm. (B,B') Controls for ablations shown in Fig. 3F: Myosin-II intensity and junction length. (B) Quantification of Myosin-II fluorescence intensity (f.i.) using the Sqh-GFP signal at ablated control, endogenous PSB and ectopic PSB junctions. Error bars show mean±s.d. Comparisons from a one-way ANOVA: DV controls vs. PSBs: p=0.0009***; DV controls vs. ectopics: p<0.0001****; PSBs vs. ectopics: p=0.0615, n.s. (B') Junction lengths of the same ablated junctions. Error bars show mean±s.d. Comparisons from a one-way ANOVA: DV controls vs. PSBs: p=0.661, n.s.; PSBs vs. ectopics: p=0.322, n.s. (C-E") Formation of ectopic PSBs in embryos expressing *UAS-wg* under the control of *rho-Gal4* (*rho>wg*). (C) Diagram showing position of deep folds at ventral ectopic PSBs in *rho>wg* embryos. (D) SEM showing short ventral folds at ectopic PSBs in *rho>wg* embryos (close-up in D'). Scale bar: 100µm. (E,E') *rho>wg* embryos immunostained against (E) Sqh1P and (E') merged with Engrailed (En). (E") Tracings of the endogenous and ectopic PSBs. Scale bar: 10µm. (F-H") Formation of ectopic PSBs in nkd^2 null mutant embryos. (F) Diagram showing position of deep folds at ectopic PSBs in nkd^2 embryos. (G) SEM showing deep folds at ectopic PSBs in nkd^2 embryos (close-up in G'). Scale bar: 100µm. (H,H') nkd^2 embryos immunostained against (H) Sqh1P and (H') merged with En and DE-Cad. (H") Tracings of endogenous and ectopic PSBs. Scale bar: 10µm. Open arrowheads depict endogenous PSBs; filled arrowheads ectopic PSBs.

Development • Supplementary information

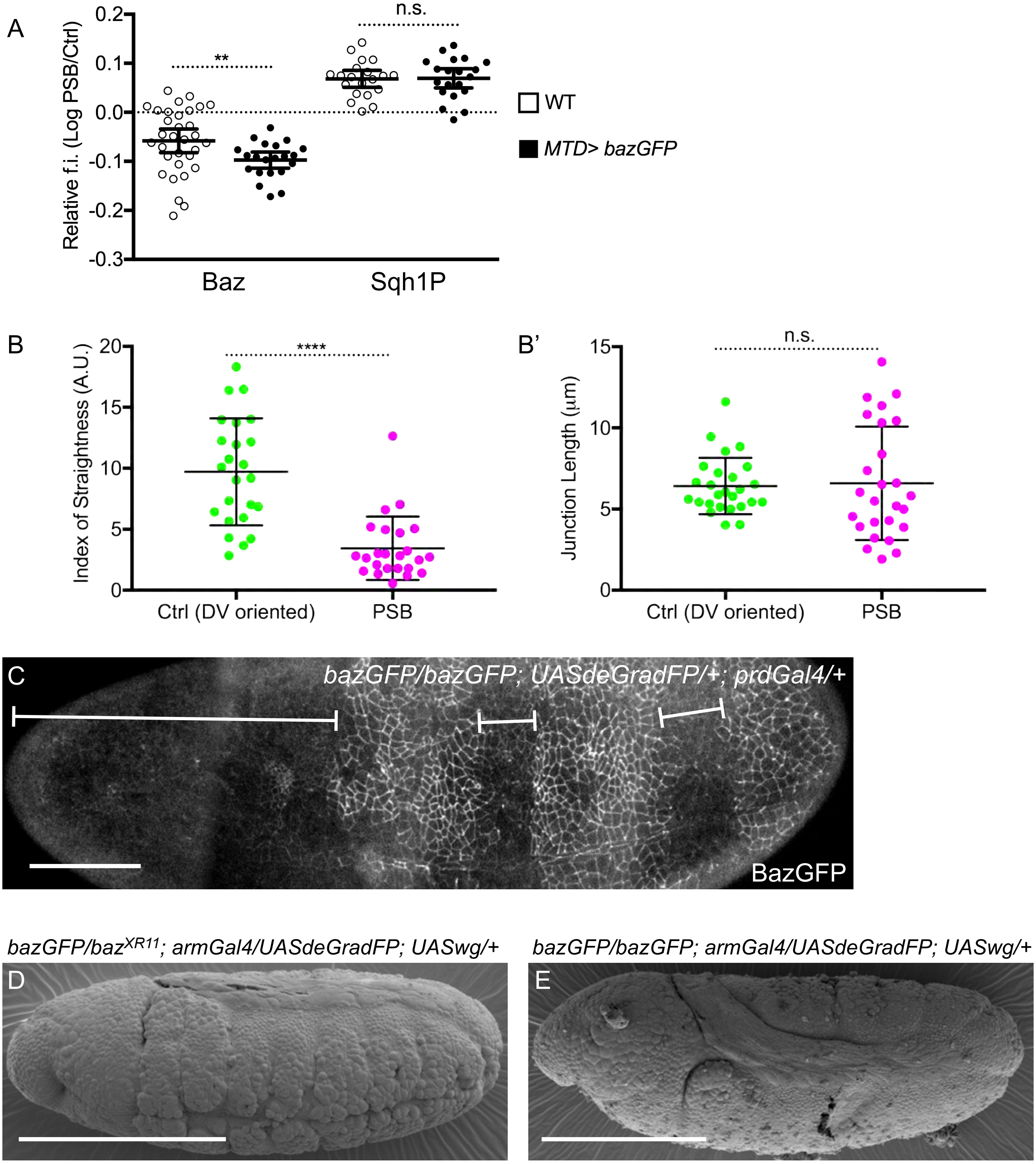

Development • Supplementary information

Figure S4: Planar polarities and actomyosin contractility at the PSBs in Bazooka-overexpressing embryos. (A) Quantification of fluorescence intensity (f.i.) of Baz and Sqh1P at PSBs relative to control DV-oriented interfaces in wildtype (open circles) and *MTD>bazGFP* (filled circles) stage 10 embryos, as $\log_{10}$. Error bars show mean±95% c.i. Baz in WT: n=32 boundaries; Baz in *MTD>bazGFP*: n=21; Sqh1P in WT: n=20; Sqh1P in *MTD>bazGFP*: n=20. Comparisons from t-tests: Baz: p=0.0081**; Sqh1P: p=0.933, n.s. (B,B') Index of straightness and junction length measurements for the laser ablations in *MTD>bazGFP* embryos (see Fig. 4C). (B) Quantification of index of straightness, a proxy for junctional tension. For both DV controls and PSBs n=24. Comparison from a Mann-Whitney test: p<0.0001****. (B') Lengths of ablated junctions at PSBs and DV controls. For both DV controls and PSBs n=26. Comparison from a Mann-Whitney test: p=0.528, n.s. Error bars show mean±s.d. (C) Ventral view of a live stage 10 embryo expressing BazGFP, with degradation of GFP-tagged protein due to expression of deGradFP under the control of *prdGal4*. Brackets show loss of BazGFP signal in *prd-Gal4* domains. Scale bar: 50µm. (D,E) SEM of embryos depleted for Baz by deGradFP in *arm>wg* embryos. Scale bars: 100µm. (D) A sensitized *baz* genetic background with *bazGFP/baz*^{XR11} in addition to deGradFP under the control of *armGal4* causes loss of epithelial integrity, especially ventrally (cells are rounding up), but deep folds remain. n=18 embryos. (E) In an embryo with *bazGFP/bazGFP* with deGradFP under the control of *armGal4*, loss of epithelial integrity is less pronounced. n=6 embryos.

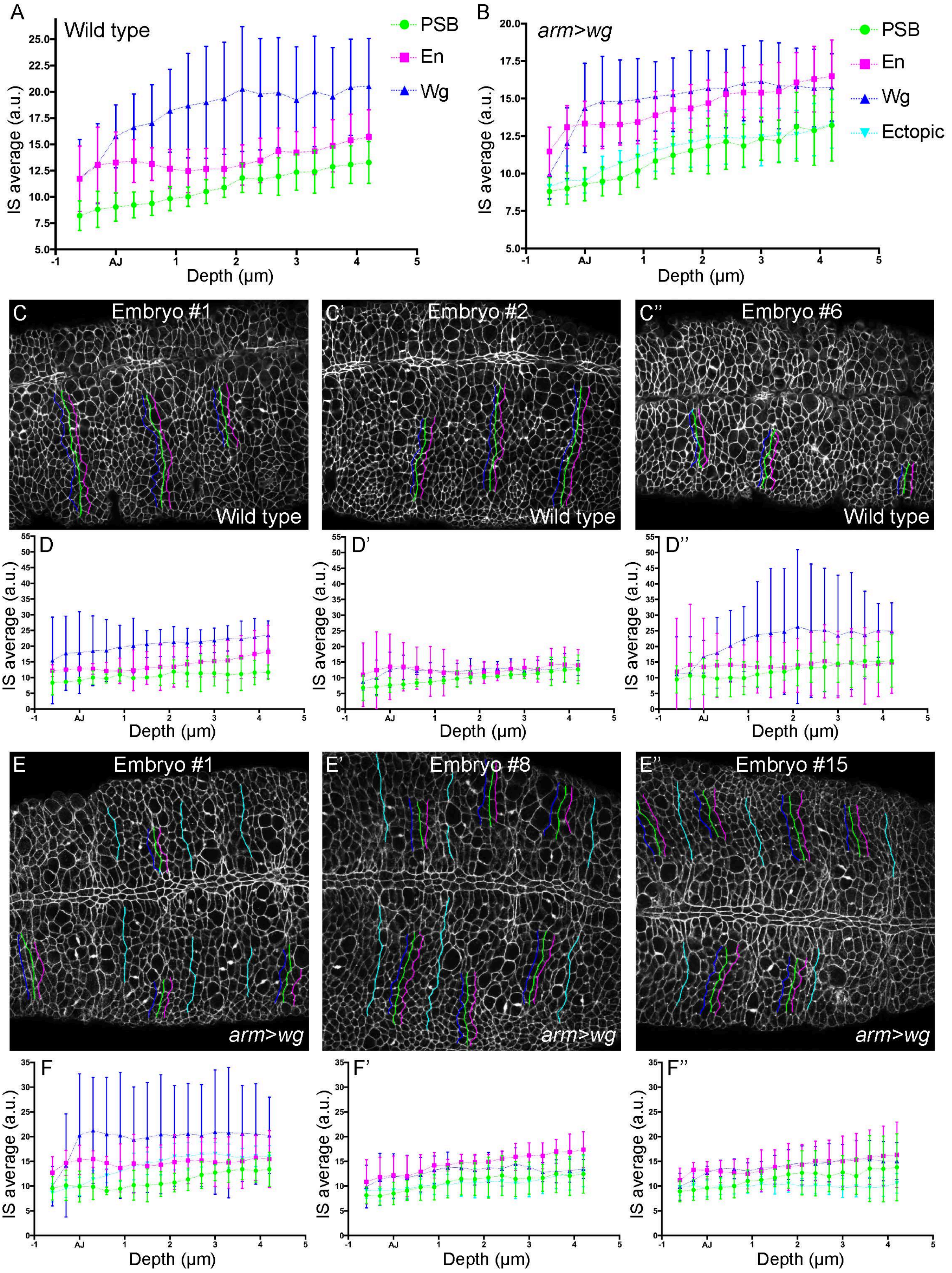
A
Wild type
PSB
En
Wg
IS average (a.u.)
Depth (µm)
B
arm>wg
PSB
En
Wg
Ectopic
IS average (a.u.)
Depth (µm)
C
Embryo #1
Wild type
C'
Embryo #2
Wild type
C''
Embryo #6
Wild type
D
D'
D''
IS average (a.u.)
Depth (µm)
E
Embryo #1
arm>wg
E'
Embryo #8
arm>wg
E''
Embryo #15
arm>wg
F
F'
F''
IS average (a.u.)
Depth (µm)
AJ

Figure S5: Index of straightness for endogenous and ectopic PSBs above and below adherens junctions. (A,B) Plots of the average index of straightness (IS) measured at different positions along the apicobasal axis (relative to the adherens junctions, AJ) in (A) wildtype and (B) *arm>wg* embryos, for PSB traces (green), control traces one cell away toward the posterior (En side, magenta), control traces one cell toward the anterior (Wg side, blue), and traces along the ectopic PSBs (cyan). n=3 embryos of each type. Error bars show mean±95% c.i. (C-C”) Confocal images showing the three wildtype embryos used in (A), as a single Z slice from the phalloidin staining, overlaid with the positions of the traces used to measure IS. (D-D”) Plots of average IS vs. apicobasal depth for each embryo in (C-C”), respectively. (E-E”) Confocal images showing the three *arm>wg* embryos used in (B), as a single Z slice from the phalloidin staining, overlaid with the positions of the traces used to measure IS. (F-F”) Plots of the average IS vs. apicobasal depth for each embryo in (E-E”), respectively. Error bars show mean±95% c.i. Note that the same 6 embryos are used in Fig.7.

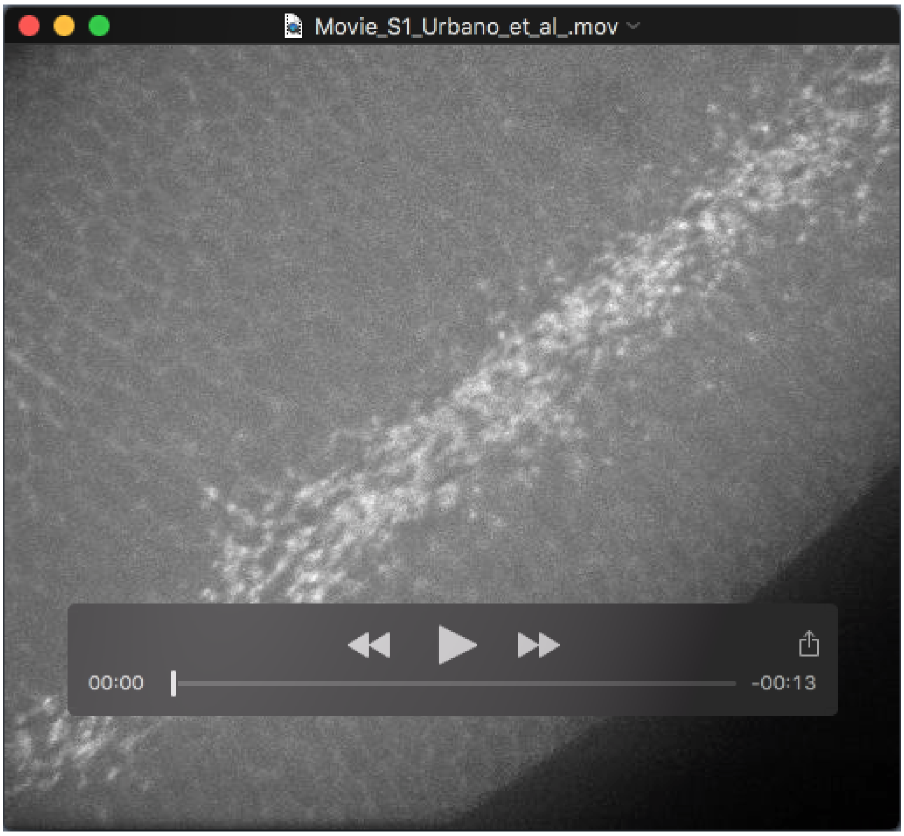

Movie 1: Myosin-II-like localisation of Flw-YFP during mesoderm invagination and early germband extension. Flw-YFP can be seen in the apices of presumptive mesoderm cells, and is present in the medial pulsatile flows at the apical cortex of cells in the extending germband, as well as being planar polarised at their junctions.

Development • Supplementary information
